# Dynamic basal ganglia output signals license and suppress forelimb movements

**DOI:** 10.1038/s41586-025-09066-z

**Published:** 2025-05-28

**Authors:** Antonio Falasconi, Harsh Kanodia, Silvia Arber

**Affiliations:** 1 Biozentrum, University of Basel, Basel, Switzerland; 2 Friedrich Miescher Institute for Biomedical Research, Basel, Switzerland; 3 Aligning Science Across Parkinson’s (ASAP) Collaborative Research Network, Chevy Chase, MD USA

**Keywords:** Neural circuits, Basal ganglia

## Abstract

The basal ganglia are fundamental to motor control and their dysfunction is linked to motor deficits^1–8^. Influential investigations on the primate oculomotor system posited that movement generally depends on transient pauses of tonically firing inhibitory basal ganglia output neurons releasing brainstem motor centres^9,10^. However, prominent increases in basal ganglia output neuron firing observed during other motor tasks cast doubts on the proposed mechanisms of movement regulation through basal ganglia circuitry^11–22^. Here we show that basal ganglia output neurons in the mouse substantia nigra pars reticulata (SNr) represent complex forelimb movements with highly granular and dynamic changes in spiking activity, tiling task execution at the population level. Single SNr neurons exhibit movement-specific firing pauses as well as increases, each occurring in concert with precise and different forelimb movements. Combining optogenetics and simultaneous recordings from basal ganglia output and postsynaptic brainstem neurons, we reveal the functional role of these dynamic firing-rate changes in releasing and suppressing movement through downstream targets. Together, our results demonstrate the existence and function of highly specific and temporally precise movement representations in basal ganglia output circuitry. We propose a model in which basal ganglia output neurons fire dynamically to provide granular and bidirectional movement-specific signals for release and suppression of motor programs to downstream circuits.

## Abstract (web summary)

Basal ganglia output neurons fire dynamically in bidirectional and movement-specific patterns to license forelimb movements.

## Main

Forelimb movements allow limbed vertebrates to perform a series of life-supporting actions, such as guiding their hands to reach targets and obtain food. They are highly complex, entailing the coordination of many muscles and can be concatenated in flexible configurations depending on needs. Their generation requires precise neuronal activity patterns to unfold across different brain regions^7,8,23–26^. The basal ganglia take centre stage in this process, integrating inputs from the cortex, thalamus and dopaminergic neurons and sending outputs to the brainstem and thalamus^27^. In both primates and rodents, basal ganglia lesions or focal perturbations degrade skilled forelimb control^1–5,7,8^ and neurological disorders of basal ganglia circuits, like Parkinson’s disease or chorea, can either impair or abnormally recruit forelimb movement^6^. Despite this functional evidence, how neuronal activity within the basal ganglia contributes to forelimb control has remained unclear.

The SNr is the main rodent basal ganglia output nucleus consisting of subpopulations of inhibitory neurons, each innervating specific motor centres in the brainstem and subdivisions of the thalamus^27–30^. SNr neurons fire at high baseline rates that, according to the textbook model, keep downstream targets under constant inhibition, with short-lived decreases in firing rate (that is, pauses) allowing movement execution through the transient release of tonic inhibition^9,10,17,24,31^. This model is largely based on work in the primate oculomotor system, studying the execution of horizontal eye movements, with limited degrees of freedom. However, recording SNr neurons during nose poking and licking revealed not only firing decreases but also bidirectional modulation with increases^22^, and recordings from basal ganglia output neurons during more complex forelimb tasks or postural changes revealed the prevalence of firing-rate increases in relation to movement^11–18,20^, together casting doubts on the general applicability of the disinhibition model of action selection. Specifically, whether decreases and increases in spike rate are properties of the majority of SNr neurons and the extent to which these firing-rate changes are related to fine-grained movements have remained unclear. In fact, functional models of basal ganglia circuits posit that, as a movement is selected, other competing motor programs are suppressed^6,32^. However, how such a selection–suppression system might be implemented at the neuronal level through basal ganglia output is unclear.

Brain circuits regulating forelimb movement outside the basal ganglia include the cortex, thalamus and brainstem^8,23,26,33–35^. In rodents, a region in the caudal brainstem referred to as the lateral rostral medulla (latRM) contains neuronal populations with different descending projection patterns and forelimb control roles^26^. It receives cortical input that is highly organized anatomically and functionally^35^, as well as projections from the caudal and lateral part of the SNr^29,30^. This SNr domain has long been hypothesized to contribute to forelimb control because of inputs received from forelimb areas of the striatum^36,37^. However, its neuronal activity dynamics and contribution to forelimb movement have remained unexplored.

Here we identify neuronal correlates of forelimb control in the SNr, exploiting the latRM as an anatomical entry point. Through electrophysiological recordings and optogenetic manipulations, we reveal how precisely timed firing decreases and increases of SNr neurons contribute to forelimb movement specification through disinhibition and suppression of downstream targets, respectively, aligning to executed movements with a high level of granularity. Our findings demonstrate that individual basal ganglia output neurons dynamically shape motor program execution, disinhibiting downstream neurons for the production of one movement and suppressing them as other specific movements are generated, thereby using a fine-grained and bidirectional encoding of individual movements.

## Diverse SNr responses during forelimb movement

**Fig. 1:**
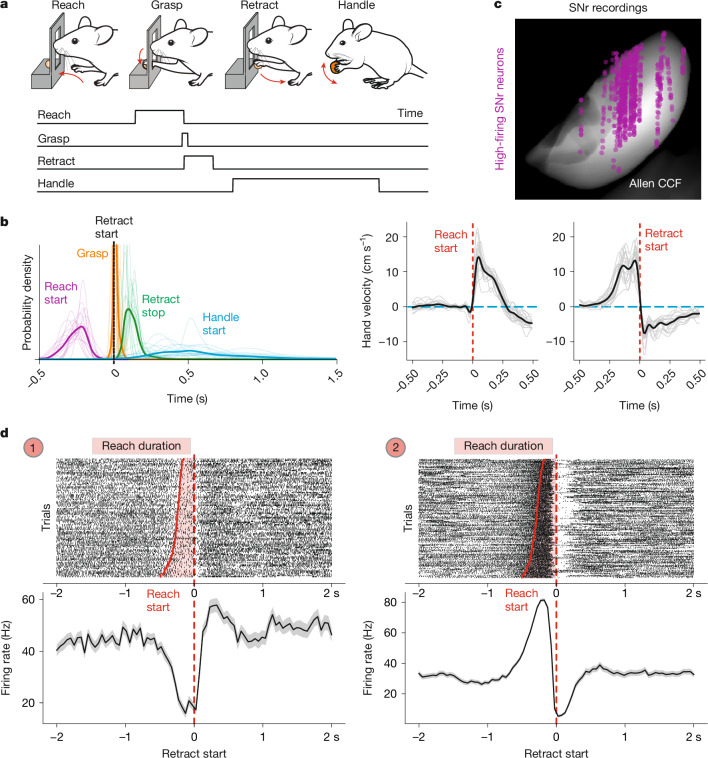
Diverse SNr neuronal responses during the forelimb task. **a**, Schematic and ethogram of forelimb movements executed in the food-pellet-reaching assay: reach, grasp, retract and handle. **b**, The probability density of the relative timing of task events across single mice (thin lines) and on average (thick lines) (left); and the average hand velocity (black line) across all of the recorded mice (*n* = 17, grey lines) aligned to reach start (middle) and retract start (right). **c**, The location of recorded SNr single units (*n* = 646) with a baseline firing rate of greater than 5 Hz in an anteroposterior projection of the SNr aligned to Allen CCF space. Top is dorsal and right is lateral. **d**, Single-trial raster plots (top; trials are sorted by reach duration) and the perievent mean ± s.e.m. firing rate (bottom) of two SNr single units aligned to retract start. Neuron 1 displays a pause in firing aligned to the onset of reach and persisting during movement execution. Neuron 2 increases in firing at reach start and pauses for execution of forelimb retraction. Note the reliability across multiple trials of the same movements as seen in single-trial raster plots. Source Data

To determine whether SNr neurons exhibit forelimb movement-related firing-rate changes, we recorded their activity in freely moving mice that were trained to perform a food-pellet retrieval task. This entailed forelimb reaching through a slit, grasping of the pellet, retraction of the limb towards the body and food handling (Fig. 1a,b and Supplementary Video 1). These movements occurred in sequence with variable timing across trials, in the range of hundreds of milliseconds for reaching and retraction and seconds for handling start, while grasping of the pellet was time-locked to the onset of retraction (Fig. 1b). Notably, the relative variability in the timing of task events was consistent across mice and markerless tracking of the reaching hand revealed stereotyped velocity profiles across mice during both reaching and retraction (Fig. 1b). This setup therefore enabled us to leverage the attributes of variability in task timing and consistency across mice to carefully probe the relationship between movement and neuronal encoding.

**Extended Data Fig. 1:**
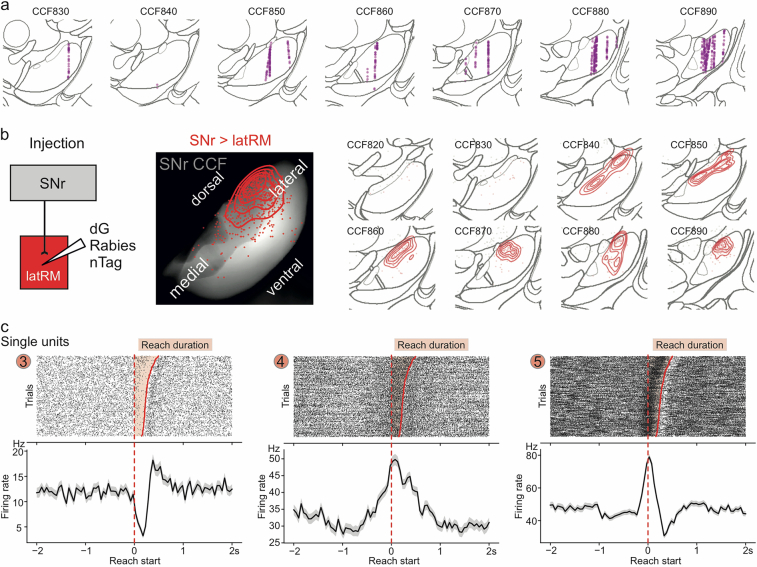
Location and modulation of recorded SNr neurons. **a**, Allen Common Coordinate Framework (CCF) location of recorded SNr single units with baseline firing rate greater than 5 Hz. Sections are spaced 100 micrometres (CCF levels). Top is dorsal and right is lateral. **b**, Schematic of viral injection for retrograde labelling of latRM-projection neurons in SNr (G-deleted Rabies expressing a fluorescent protein localized to the nucleus (nTag)) (left). Location of retrogradely labelled nuclei (from n = 4 mice) in an anteroposterior projection of the SNr (centre) and in single CCF levels (right). Lines represent isodensity lines computed using kernel density estimation. Top is dorsal and right is lateral. **c**, Single trial raster plots (top) and average firing rate with standard error of the mean (bottom) of SNr neurons aligned to reach start (these are additional examples to the two units shown in Fig. 1). Neuron 3 displays a pause in firing during reaching. Neuron 4 increases in firing around reach start. Neuron 5 displays both an increase (during reach) and a pause (during retract). Note the diverse activity patterns in different single units. Source Data

To measure SNr activity, we implanted Neuropixels probes in trained mice (Fig. 1c and Methods; 646 tonically active neurons across 17 mice). We aimed to target the caudal and lateral portion of the nucleus (Fig. 1c and Extended Data Fig. 1a), containing neurons projecting to the latRM (Extended Data Fig. 1b), a forelimb control centre in the caudal brainstem^26^. Inspection of single-trial neuronal activity and perievent time histograms (PETHs) for single SNr neurons revealed dynamic firing-rate increases and decreases (Fig. 1d and Extended Data Fig. 1c). These findings underscore the complexity of SNr neuron activity during a flexibly executed forelimb movement sequence, displaying both firing pauses and increases with multiphasic activity patterns. Our observations call for a careful dissection of these dynamic firing-rate changes aligned to precise movements, described in the following sections.

## Granular movement tuning of SNr neuronal dynamics

**Fig. 2:**
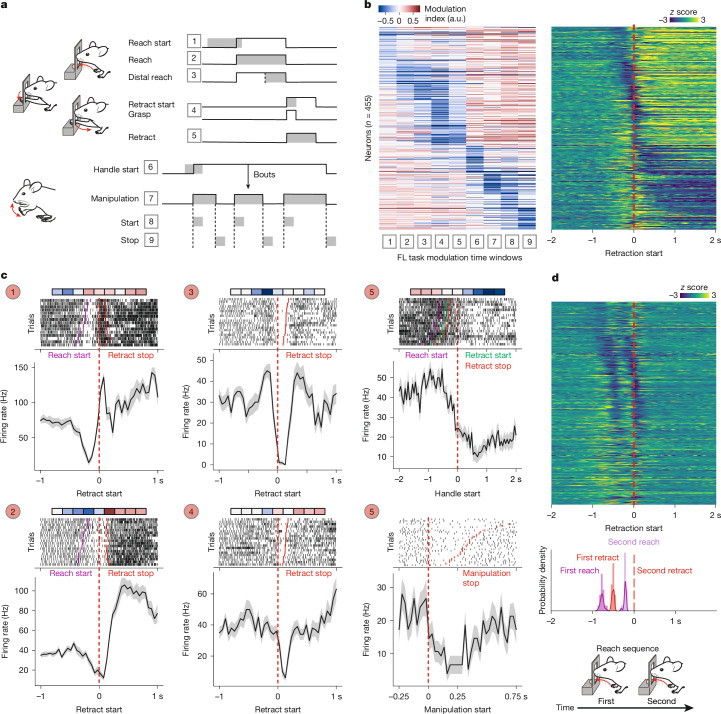
Granular movement tuning of SNr firing changes. **a**, The task time windows under consideration to estimate neuronal firing-rate changes during the forelimb task. Time windows are represented as grey shaded areas overlaid on task events in black lines and numbered 1–9. **b**, The computed task time window modulation (left) and *z*-scored average firing rate (right), aligned to the onset of retraction, of SNr neurons with negative task modulation (*n* = 455). Neurons were sorted on the basis of their window of maximum negative modulation in the modulation heat map. Note that decreases in spike rate tile movement execution and are accompanied by increases in firing in single neurons during task windows different from the decrease-related ones. **c**, Single-neuron modulation to task time windows (top; the sequence of boxes from left to right corresponds to time windows 1–9 as in **b** with the same colour scale for modulation indices), single trial raster plots (middle) and the perievent mean ± s.e.m. of the firing rate (bottom) of five SNr neurons aligned to the timepoints indicated on the *x* axis, and with ticks displaying select other task events in raster plots as indicated. Each of the neurons displays precise pauses and increases in firing aligned with different task events. **d**, The *z*-scored average firing rate of negatively modulated SNr neurons sorted as in **b**, aligned to the second retraction start (top). Middle, the median occurrence times of the first reach, first retraction and second reach relative to the second retraction (red dashed line) across mice. Bottom, schematic of reach repetition trials in which two reaches were executed within 0.3–0.6 s. Pauses in single SNr neurons that are negatively modulated during reach and retraction repeat twice as these movements are executed twice in sequence. Note the absence of handling-related pauses in the bottom part of the heat map, given the absence of pellet retrievals in these trials. Source Data

**Extended Data Fig. 2:**
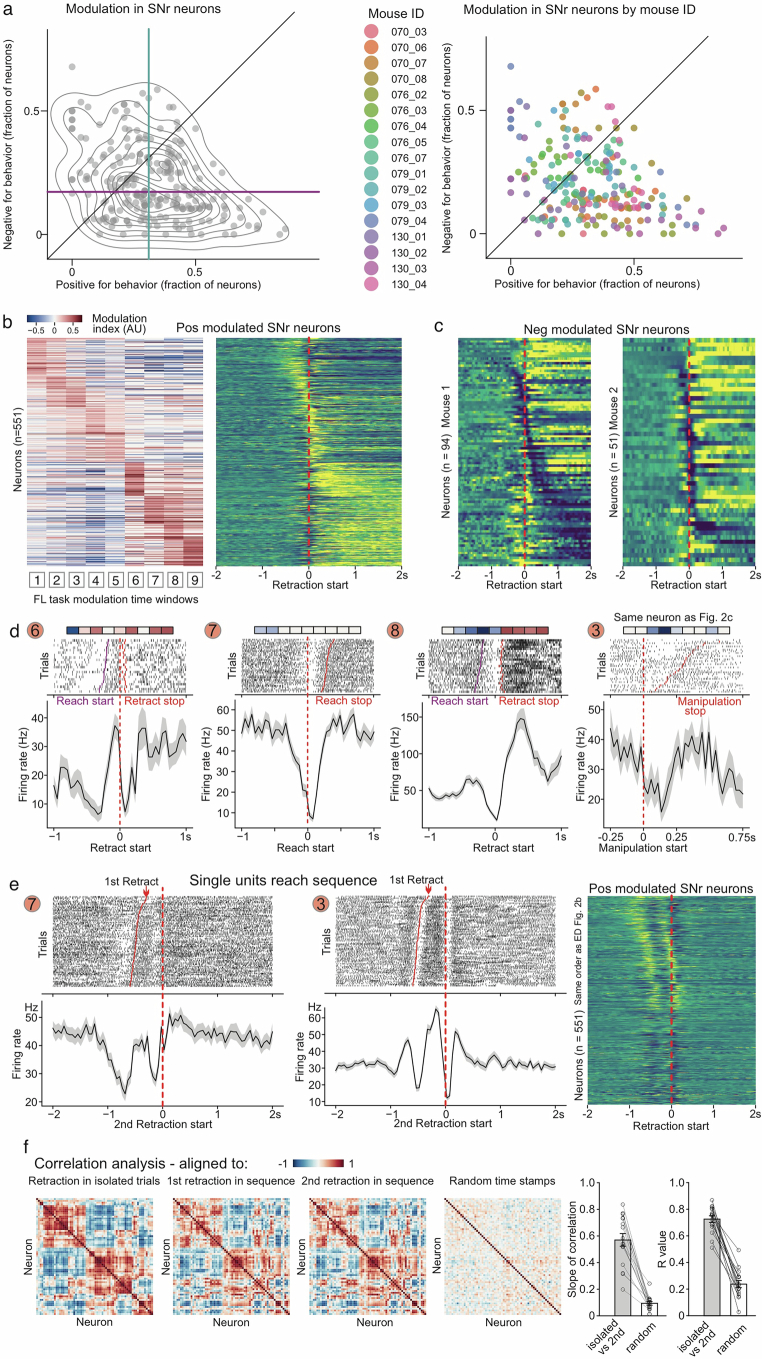
Granular movement tuning of SNr firing changes. **a**, Scatter plot displaying the fraction of positively versus that of negatively modulated neurons for each task time window (Fig. 2a) across the dataset (left) and in each single mouse (right). The median fraction of positively modulated units (cyan vertical line) is higher than that of negatively modulated units (magenta horizontal line) for each behaviour and across mice. **b**, Heatmaps displaying the computed task time window modulation (left) and z-scored average firing rate aligned to the onset of retraction of SNr neurons with positive task modulation (right). Neurons were sorted based on their window of maximum positive modulation in the modulation heatmap. Note that increases in spike rate in heatmap (yellow) tile movement execution. **c**, Heatmaps displaying the z-scored average firing rate of SNr neurons gathered from 2 single mice (mouse 1 and 2), with negative task modulation aligned to the onset of retraction and sorted by trough time. Note decreases in spike rate (blue) tiling movement execution similar to the full dataset across all mice. **d**, Single neuron modulation to task time windows (top; color code and sequence of boxes as in Fig. 2b), single trial raster plots (middle) and average firing rate with standard error of the mean (bottom) of SNr neurons (additional examples to the three units shown in Fig. 2, except for rightmost plot aligned to manipulation start) aligned to reach start, retract start or manipulation start. Each of the neurons displays a very precise pause in firing aligned with specific task time windows. Note for neuron 3, its negative modulation during manipulation, paralleling its pause aligned to retraction start. **e**, (left) Single trial raster plots (top) and average firing rate with standard error of the mean (bottom) of the same SNr neurons (7) and (3) as displayed in Fig. 2a, Extended Data Fig. 2d, aligned to the second retraction start in trials where two reaches were executed in a 0.3-0.6 s interval. The pauses in firing repeat twice as the movements are executed twice. (right) Heatmap displaying the z-scored average firing rate aligned to the onset of retraction of SNr neurons with positive task modulation. Neurons were sorted as in Extended Data Fig. 2b. Note that increases related to reaching and retraction repeat twice as movements are executed twice while handling-related spiking rate changes are absent since mice do not collect the food pellet in these trials. **f**, (left) Heatmaps displaying pairwise correlation of average neuronal activity of tonically firing SNr neurons from one mouse in 4 s windows around retraction start in isolated trials (left), first retraction start in dual reach trials (centre-left), second retraction start in dual reach trials (centre-right) and 100 random timestamps (right). (right) Bar plot (mean and standard error) displaying the correlation slope and R value resulting from regressing the correlation matrix for isolated retraction start and second retraction start in dual reach trials compared to those resulting from correlating the correlation matrix for isolated retraction start and random timestamps (n = 17 mice). Note preservation of correlations in neuronal population activity when aligning to retraction in isolated trials or upon repetitions of the movement and their disappearance when considering random timestamps indicating the movement-related nature of these firing patterns. Source Data

To determine the granularity of movement representation through SNr firing changes, we quantified firing-rate modulation of SNr neurons during distinct time windows in the forelimb task, encompassing reach–grasp–retrieval movements as well as movements related to food handling and manipulation^38^ (Fig. 2a and Methods). We found that 629 out of 646 of the recorded SNr neurons were modulated during these nine defined task windows. In total, 72% of SNr neurons were negatively modulated to at least one task window and 88% displayed positive modulation, with the fraction of positively modulated neurons being consistently higher than that of pausing units for behavioural events analysed and across mice (Extended Data Fig. 2a), in agreement with previous reports^19^. Notably, 60% of the modulated units displayed bidirectional modulation during the task, that is, they decreased their firing during at least one examined time window and increased during at least another, suggesting that SNr neurons pause their spiking activity during the performance of select movements and increase in firing as other movements are executed.

We next visualized the modulation of negatively and positively modulated SNr neurons to each of the different time windows, together with their trial-averaged *z*-scored firing rates aligned to the retraction start. We sorted neurons on the basis of the task time window during which they displayed the highest modulation (Fig. 2b and Extended Data Fig. 2b). Precisely timed firing changes of single SNr neurons tiled the execution of the task for both decreases and increases, a finding that was also observed for SNr neurons recorded simultaneously within single mice (Extended Data Fig. 2c). Notably, increases in the firing rate in windows other than decrease-related ones were evident across the dataset (Fig. 2b), confirming the pervasive bidirectional modulation of SNr neurons during forelimb movements.

At the level of single neurons, granular and distinctive bidirectional patterns of modulation to the examined movement windows were widespread (Fig. 2c and Extended Data Fig. 2d). Across single trials, neurons displayed reliable decreases and increases in spiking activity, precisely aligned to select task events. For example, two SNr neurons (Fig. 2c; neurons 1 and 2) both decreased their firing during reaching but one of them was also negatively modulated at the reach start and increased its spiking at the retraction start, while the other paused throughout reaching and retraction but then increased its activity during handling movements. For another set of two SNr neurons (Fig. 2c and Extended Data Fig. 2b; neurons 3 and 4) both pausing firing during the retraction time windows, only one was also negatively modulated during food manipulation. Yet another SNr neuron (Fig. 2c; neuron 5) decreased its spiking during food handling and manipulation, while increasing firing during reaching-related windows.

To further probe the notion that dynamic firing patterns of single SNr neurons relate to executed movements, we took advantage of the fact that mice occasionally extended their arm through the slit twice in very quick succession (within 0.3 to 0.6 s) (Fig. 2d and Methods). We visualized the PETHs of single neurons in these repetitive-reach trials and noted an almost identical repetition of the same firing-rate modulation as movements are executed twice in sequence (Extended Data Fig. 2e). The *z*-scored mean firing of all pausing and increasing neurons in these reach repetition trials confirmed the duplication of reach- and retraction-related changes in firing rate (Fig. 2d and Extended Data Fig. 2e), occurring in the same temporal sequence as in isolated reach trials (Fig. 2b and Extended Data Fig. 2b). As a further quantification of this phenomenon, neuron-to-neuron correlations in average firing were largely similar in isolated trials and repetition trials, while they were completely abolished when aligning to random timestamps (Extended Data Fig. 2f). Moreover, inspection of the mean firing rates across all task-pausing neurons revealed the absence of handling-related pauses and increases after retraction (Fig. 2d and Extended Data Fig. 2e). In fact, repetitive-reach trials were not followed by handling due to the lack of pellet retrieval, explaining the absence of handling-related firing-rate changes compared with single-reach trials followed by pellet retrieval and handling (Fig. 2b and Extended Data Fig. 2b).

Together, these data provide strong evidence that the mapping between SNr neuronal activity and movement is fine-grained and that single SNr neurons dynamically modulate their firing rate through decreases and increases as different forelimb movements are executed.

## SNr neuronal dynamics parallel movement variation

**Extended Data Fig. 3:**
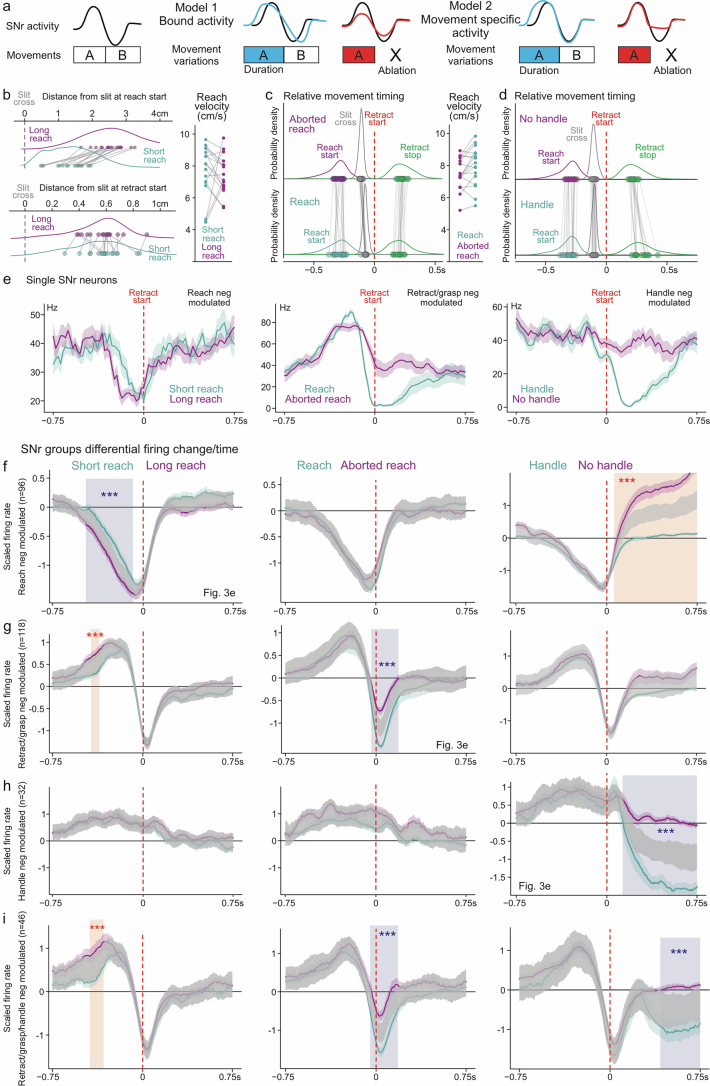
Movement-contingent SNr firing changes. **a**, Alternative hypotheses for multiphasic modulation of SNr neurons in the form of schematic neuronal activity traces. As movements are executed (A and B), consider a bidirectionally modulated neuron increasing for movement A and pausing its firing subsequently as movement B is executed (left). In Model 1 (middle), as the duration of movement A is altered (e.g. duration), the entire dynamical activity pattern could change, representing a different bound action. Similarly, when movement A is executed without movement B (ablation), the entire dynamical activity pattern could be altered to reflect the difference in the bound action or the different phases of the activity could occur similarly as if the activity pattern were temporally bound and the sequence of activity changes represented a bound movement sequence. Alternatively, in Model 2 (right), each of the phases of the activity pattern would be linked to the execution of particular movements, and hence, specifically increasing the duration of movement A would widen the activity phase linked to the execution of the movement A and leave the modulation during an unaffected movement B unchanged. Similarly, when movement B is specifically ablated from the sequence of movements being executed, and A is unchanged, the pattern of neural activity linked to the execution of movement B would be absent, while leaving dynamics related to movement A unchanged. **b**, Median distance from the slit at the start of reach (top) and start of retraction (bottom) for long and short duration reaches (n = 17 mice), and their probability density reflecting no structured covariation between the distance from slit and reach durations taken across mice (left). Comparison of median reaching velocity for short and long duration reaches revealed no consistent trend in reaching hand speed across mice (n = 17) (right). **c**, Relative median timing of task events and relative probability densities for aborted vs complete reaches revealing similar probability densities of reach starts (left) and similar velocities of the reaching hand (right) for both trial types across mice (n = 17). **d**, Relative median timing of task events and relative probability densities for reaches followed by handling and those without handling revealing similar probability densities of reach starts for both trial types across mice (n = 17). **e**, Perievent time histograms of three example SNr neurons (left, middle and right) aligned to retraction start on respective movement variation trials in cyan and magenta (short and long reach, reach and aborted reach, handle and no handle trials). **f-h**, Z-scored average firing rate of the three SNr neuronal populations aligned to retract start in the movement variation trials in cyan and magenta, overlaid with 99.9% confidence interval of the distribution of average firing rates computed over shuffled trial pairs in grey: short and long reach (left), reach and aborted reach (middle), handle and no handle trials (right). Shaded rectangles represent intervals of significant difference from the null distribution. Note, in addition to the changes in movement related pauses in the respective variations, the same neuronal populations increase in firing in a movement dependent manner as select other movements are executed. **i**, Z-scored average firing rate of the SNr neuronal population negatively modulated during retraction/grasping/handling aligned to retract start on respective movement variation trials in cyan and magenta, overlaid with 99.9% confidence interval of the distribution of average firing rates computed over shuffled trial pairs in grey: short and long reach (left), reach and aborted reach (middle), handle and no handle trials (right). Shaded rectangles represent intervals of significant difference from the null distribution. Note the increase in firing rate of this population of neurons during reaching showed an increase in its duration as the duration of the reach was increased, while the other phases of the modulation were unaffected. Similarly, the modulation at the onset of retraction was significantly different between the complete and abbreviated reaches as described in Fig. 3. Further, the negative modulation during handling was also contingent on whether the mice handled the food pellet subsequent to reaching. Source Data

The multiphasic spiking dynamics of single SNr neurons raise the question of whether they, as a whole, contribute to the production of an invariantly bound sequence of movements^39^. Alternatively, single components of the multiphasic task responses (for example, each pause and increase in spiking) could regulate sequential movements individually. Envision a movement sequence A–B, accompanied by an SNr neuron increasing activity during movement A and decreasing activity during movement B (Extended Data Fig. 3a). In the two scenarios, a prolongation in the duration of movement A could either translate into a lengthening of the whole dynamic firing pattern or into an exclusive lengthening in duration of the firing-rate change related to movement A (Extended Data Fig. 3a). Similarly, ablation of one movement from the sequence could either be paralleled by an unchanged overall dynamic firing pattern or result in an exclusive absence of the firing-rate change related to the ablated movement (Extended Data Fig. 3a).

**Fig. 3:**
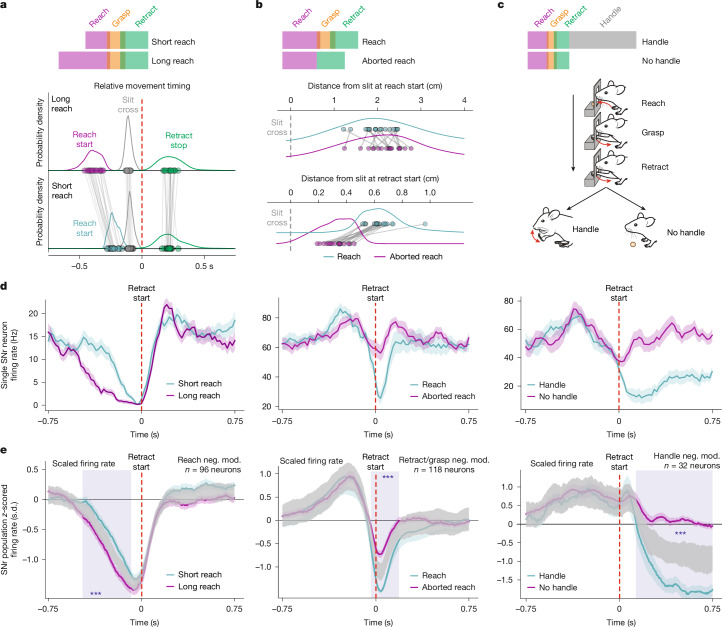
Movement-contingent SNr firing changes. **a**, Schematic of the short and long reach trials in which the reach duration was selectively altered (top), and the relative median timing of task events in the short and long reach trials (*n* = 17 mice) and their probability density (bottom). **b**, Schematic of trials in which reaching was interrupted prematurely as opposed to full reach trials entailing grasp-related movements (top), and the median distance from the slit at reach start (middle) and retract start (bottom) in reach and aborted-reach trials (*n* = 17 mice) and their probability density. **c**, Schematic of trials in which reaching, grasping and retraction were either followed by handling or not. **d**, The perievent mean ± s.e.m. of firing rate of three SNr neurons (left, middle and right) aligned to the retraction start (red dashed lines) on respective movement variation trials (cyan and magenta; short and long reach, reach and aborted reach, handle and no handle trials). **e**, The *z*-scored average firing rate ± s.e.m. of the three SNr neuronal populations (*n* = 96, 118 and 32, respectively) aligned to the retract start (red dashed lines) on respective movement variation trials (cyan and magenta), overlaid with the 99.9% confidence interval of the distribution of average firing rates computed over shuffled trial pairs (grey) (short and long reach, reach and aborted reach, handle and no handle trials). The blue shaded rectangles represent intervals of significant difference (*P* < 0.001) from the null distribution. Note the timely alterations in movement-related firing-rate changes as different movement variations occur for the three neuronal populations under study. See also Extended Data Fig. 3. Neg. mod., negatively modulated. Source Data

To disambiguate these possibilities, we took advantage of variations in the executed movement sequence (Fig. 3a,b and Supplementary Video 2). Specifically, we isolated reaches of short or long duration (Fig. 3a and Extended Data Fig. 3b), trials in which arm extension was terminated before reaching the pellet location and was therefore immediately followed by retraction (Fig. 3b and Extended Data Fig. 3c), and trials with or without food handling (Fig. 3c and Extended Data Fig. 3d). At the behavioural level, we found that trials stratified on the basis of reach duration differed in the starting location of the reach but did not display significant differences in the duration of other task events, in the mean reach velocity or in the end-point distance from the slit (Fig. 3a, Extended Data Fig. 3b and Supplementary Video 2). Trials with abbreviated reaches differed in the end-point distance from the slit and, expectedly, in the relative timing of slit crossing with respect to retraction start, but did not display consistently different reach duration, reach velocity and distance from the slit at reach start (Fig. 3b, Extended Data Fig. 3c and Supplementary Video 2). Finally, trials with or without handling did not display any difference in relative timing of reach start and slit crossing with respect to retraction start and displayed longer retraction (Extended Data Fig. 3d and Supplementary Video 2). Together, this analysis of behavioural variation data suggests that the selected trial types, across mice, reflect isolated alterations in proximal forelimb movements during reaching (Fig. 3a), distal forelimb movements executed in between reach and retraction (Fig. 3b), or the retract-to-food-handling transition (Fig. 3c).

We next addressed the impact of these three behavioural variations on SNr firing-rate changes. We isolated SNr neurons from our dataset on the basis of their negative modulation to specific groups of task time windows related to the variable behaviours analysed. Specifically, we selected SNr neurons pausing throughout arm extension (1), selectively at retraction onset (2) or at handling onset (3). Inspection of single SNr neurons from these classes across behavioural variations showed that alterations in the executed movement sequence corresponded to changes in task-related firing responses, in agreement with their measured modulation to different task time windows (Fig. 3d and Extended Data Fig. 3e). Qualitatively, SNr neurons pausing during reach time windows showed pauses of longer duration as the reach duration increased (Fig. 3d (left) and Extended Data Fig. 3e), select SNr neurons modulated around the onset of retraction did not pause their spiking as the reach was aborted before reaching the pellet location (Fig. 3d (middle) and Extended Data Fig. 3e) and handling-related responses were abolished when the mice did not handle food after reaching and retracting the forelimb (Fig. 3d (right) and Extended Data Fig. 3e).

To evaluate these effects quantitatively, we visualized the average firing rate of each neuron class aligned to retraction start in the different movement variations and compared it to a null distribution of average firing rates computed by randomly sampling trials from the two trial types (Fig. 3e, Extended Data Fig. 3f–h and Methods). Each neuronal population displayed a significant difference in its pause as the pause-related movements were specifically altered in the movement sequence (Fig. 3e and Extended Data Fig. 3f–h), in agreement with a model in which the single components of SNr neuron responses relate to the individual movements being performed (Extended Data Fig. 3a). Notably, and corroborating this idea, for the same neuron classes, alterations in movements different from the pause-related one did not correspond to changes in their firing-rate decreases but, in some cases, corresponded to changes in their firing-rate increases (Extended Data Fig. 3f–h). Specifically, the increases in firing rate during handling of neurons pausing during reaching were not present when food handling movements were not performed (Extended Data Fig. 3f). Similarly, the increase in the firing rate during reaching displayed by neurons pausing at the retraction start scaled in duration as the reach duration increased (Extended Data Fig. 3g).

Furthermore, having identified neurons that were negatively modulated in time windows related to grasping and retraction as well as during food handling and manipulation (Fig. 2a,c and Extended Data Fig. 2d), we probed whether reaches that terminated before engaging with the pellet and trials in which no food handling followed reaching and retraction altered specific components of their task-related firing dynamics (Extended Data Fig. 3i). The absence of handling movements corresponded to the absence of handling-related firing-rate decreases in this neuronal population, while the retract start aligned pause was unaltered (Extended Data Fig. 3i (right)). On the contrary, abbreviated reaching trials corresponded to a significant change in the retraction-start-aligned decrease in this neuronal population (Extended Data Fig. 3i (middle)), again confirming that each phase of the multiphasic dynamics of SNr neurons is independently regulated.

Together, these data provide support for a model in which executed movements are the main predictor of SNr neuron dynamic firing patterns, rather than modulation of SNr neurons being invariantly linked to the production of a bound action. In this model, SNr neurons would pause their spiking to release one movement and increase it to suppress that same movement when specific other movements are executed.

## SNr firing pauses are needed for forelimb movement

**Fig. 4:**
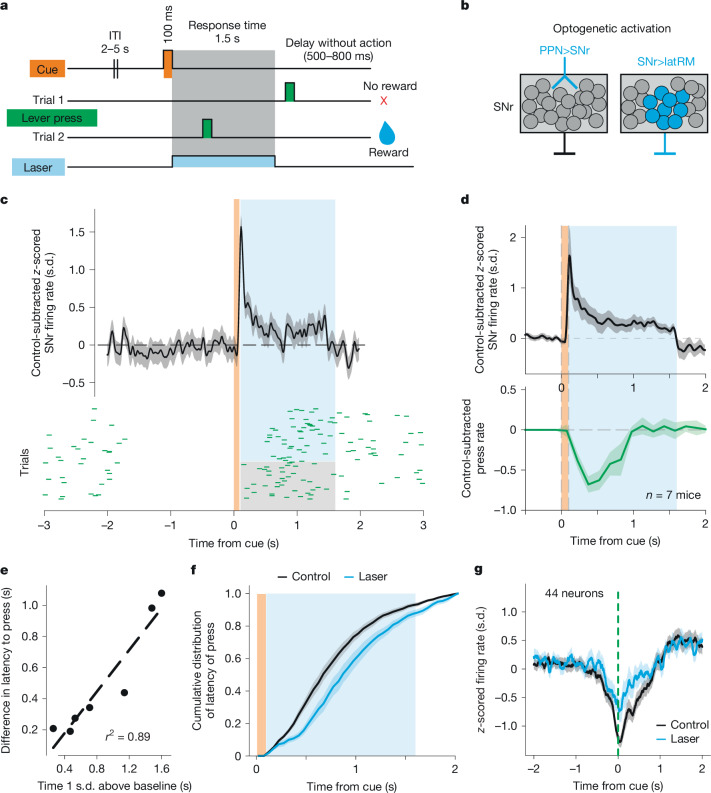
SNr firing pauses are needed for forelimb movement. **a**, The task structure, representing the cue, lever and laser state in time. After a 2–5 s intertrial interval (ITI), a visual cue is presented for 100 ms, after which the mice are required to press the lever within a 1.5 s response time. If a press is executed in the response time, a reward is delivered. On 45–50% of randomly interleaved trials, SNr neurons were optogenetically activated. **b**, The dual strategy used to optogenetically activate SNr neurons. Left, activation of excitatory projections to the SNr from the pedunculopontine nucleus (PPN) (*n* = 9), expressing the opsin ChRmine or ChR2. Right, activation of latRM-projecting SNr neurons (*n* = 5), expressing the opsin ChRmine. In the case of PPN>SNr activation, we recorded SNr neuronal activity (*n* = 257 neurons from 7 mice). **c**, The mean ± s.e.m. control-subtracted *z*-scored firing rate of SNr neurons in a single mouse (*n* = 27) on laser trials (top). Bottom, raster plot of single trials from the same mouse in control (grey shading) versus laser (blue shading) conditions; each green mark represents a lever press. **d**, The mean ± s.e.m. *z*-scored control-subtracted firing rate of recorded SNr neurons over all mice (*n* = 257) on laser trials (blue shading) (top). Bottom, the mean control-subtracted press rate (green) during laser trials (blue shading). **e**, The difference in median press latency as a function of the duration of the optogenetically evoked increase in SNr firing in single mice (*n* = 7, Pearson *R*^2^ = 0.89, one-sided Wald test, *P* = 0.0007). **f**, The mean ± s.e.m. cumulative distribution of latency to press in laser (blue) and control (grey) trials. *n* = 15 mice. **g**, The *z*-scored mean ± s.e.m. of firing rate of SNr neurons displaying pauses during press events (*n* = 44) on control trials (black) compared with laser trials (blue), shown across mice. Source Data

To address the role of SNr neurons in forelimb movement execution, we sought to perturb firing pauses through optogenetic means. As the timing of perturbation of basal ganglia circuits has proven to be critical during action selection^40^, we activated SNr neurons in mice trained to perform a cued lever-pressing task, where the intention to move is triggered by a visual cue (Fig. 4a). This enabled us to perturb SNr neurons before the onset of forelimb movement. We either optogenetically activated caudal lateral SNr neurons projecting to the latRM or glutamatergic input to the SNr from the pedunculopontine nucleus (PPN>SNr), using *Rbp4-cre* mice as a genetic entry point to these neurons^41^ (Fig. 4b). Stimulation of glutamatergic PPN>SNr neurons enabled us to simultaneously monitor the responses of SNr neurons during optogenetic perturbation. This provided a means to examine evoked changes in the firing rate and the causal relationship between activity patterns and movement.

**Extended Data Fig. 4:**
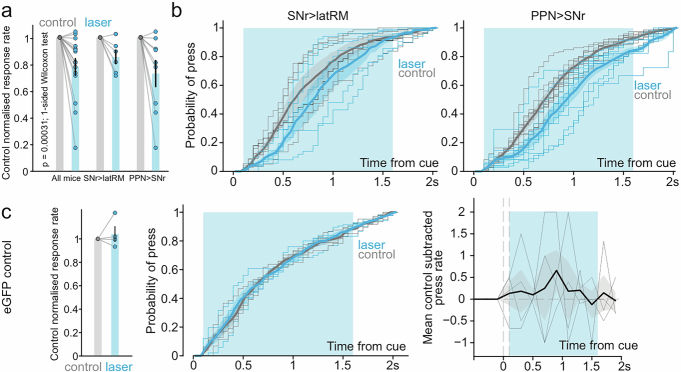
Optogenetic activation of SNr neurons delays lever pressing. **a**, Bar plot displaying the mean and standard error thereof of the control normalized response rate upon optogenetic activation of SNr neurons with the two approaches detailed in Fig. 4b. Single dots represent single mice (all mice n = 15, SNr>latRM n = 6, PPN>SNr n = 9). **b**, Average and standard error of the mean of the cumulative distribution of latency to press in laser (blue) and control (grey) trials across mice for SNr>latRM activation (left) and optogenetic activation of PPN>SNr excitatory projections. Thin lines represent single mouse data. **c**, Bar plot displaying the mean and standard error thereof of the control normalized response rate in control mice expressing eGFP in either the PPN or SNr neurons with an optic fibre implanted in the PPN or SNr respectively to control for the effects of light on the performance of the mice (n = 4) (left). Average and standard error of the mean of the cumulative distribution of latency to press in laser (blue) and control (grey) trials across all control mice (middle). No effect was observed in the press rate or the latency to press the lever in the control mice upon the exposure of the cells to the laser light. Mean control subtracted press rate (black) during laser trials (blue shade) in the control mice showed no stereotyped change in press rate across the control mice (right) over the response window. Source Data

**Extended Data Fig. 5:**
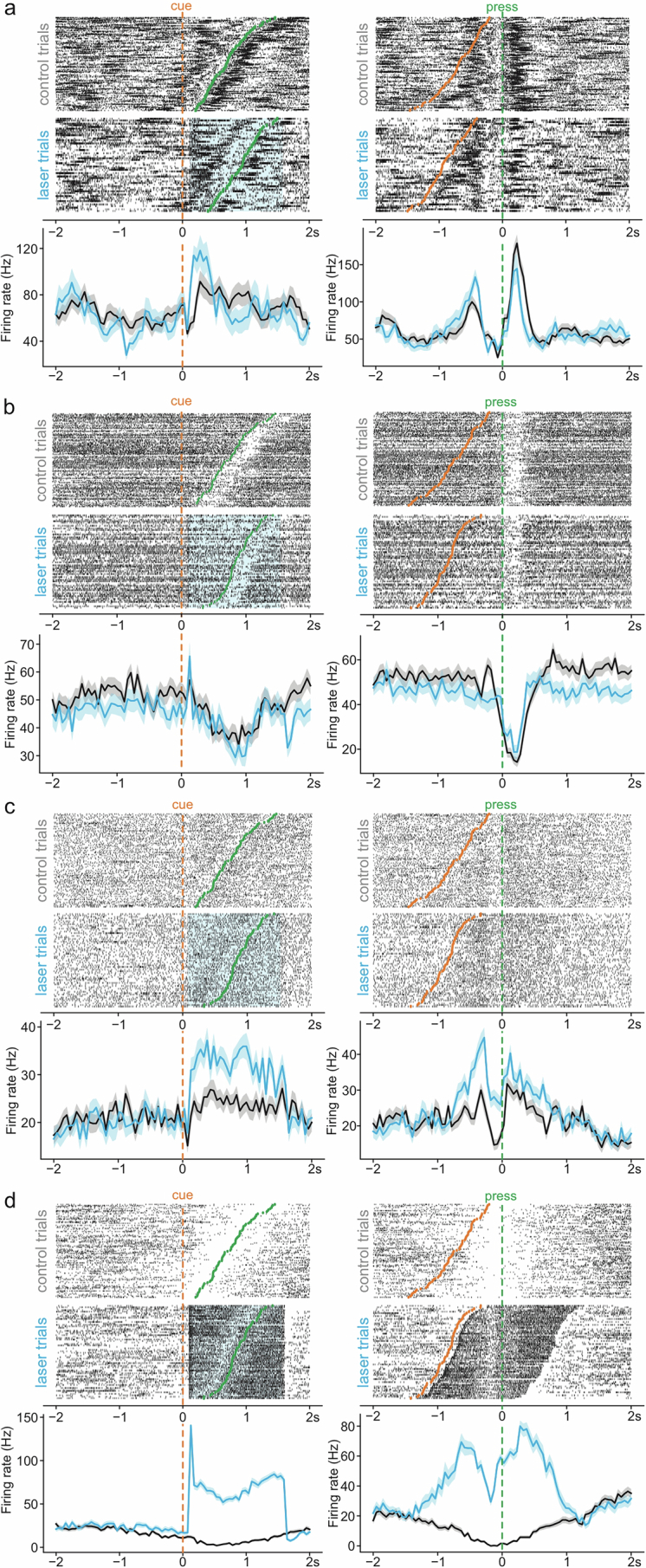
Pauses in firing of optogenetically activated SNr neurons align with behaviour. **a-d**, Single trial raster plots (top) and average firing rate with standard error of the mean (bottom) of single SNr neurons aligned to cue presentation (left) and lever press (right) on control (top, grey in average) and laser trials (bottom, blue in average) in which a press was executed within a 1.5 s interval after the cue. Note for all neurons increase in spike rate over baseline upon laser exposure (left; note the variability in single neuron responses to the optogenetic perturbation, despite the overall transient effect at the population level shown in Fig. 4d) followed by a pause in both control and laser trials aligned to lever press (right). Light blue boxes indicates periods of laser application.

We first assessed whether and to what extent optogenetic stimulation after cue offset affected firing rates of SNr neurons in mice with simultaneous perturbation and SNr recordings. Measuring the control-subtracted laser-evoked response of SNr neurons revealed that light exposure caused a transient increase in firing rate at the neuronal population level (Fig. 4c,d). Notably, the period with an increased firing rate coincided with a sharp decrease in the number of executed lever presses relative to control trials in single mice (Fig. 4c) and in decreased press rates across mice (Fig. 4d). Lever presses became more likely once the effect of stimulation on SNr firing was extinguished at the population level (Fig. 4c,d), suggesting that activation of SNr neurons is probably responsible for the observed antikinetic phenotype. In support, regressing the average duration of effective SNr optogenetic activation against the median increase in latency to press after cue presentation revealed a strong positive correlation (Fig. 4e and Methods; *R*^2^ = 0.89, *P* = 0.0007). Coherent with these findings, quantifying the effects of optogenetic perturbation across all mice, we found that stimulation led to an increase in the latency to press the lever and in the fraction of no response trials across mice (Fig. 4f, Extended Data Fig. 4a,b and Supplementary Video 3), while no such phenotype was observed in light exposure controls, expressing GFP instead of optogenetic actuators (Extended Data Fig. 4c). To further explore the causal relationship between SNr neuron activity patterns and movement, we exploited lever presses performed despite optogenetic excitation of SNr neurons. If firing pauses in select SNr neurons are needed for movement, these should occur during press execution in trials with or without optogenetic activation. To test this hypothesis, we isolated SNr neurons decreasing in firing rate around the press (Methods) and plotted their PETHs aligned to cue presentation and press execution in both stimulation and control trials. We found that the *z*-scored average firing rate of neurons pausing for press execution in control trials decreased similarly in trials in which a press was executed despite optogenetic SNr activation, suggesting that firing pauses of SNr neurons support movement execution (Fig. 4g). Corroborating this notion, firing-rate decreases aligned to press execution were evident in single SNr neurons that increased their firing rate either continuously or transiently after optogenetic excitation (Extended Data Fig. 5a–d). Together, these findings provide causal evidence for the importance of bidirectional SNr firing-rate changes in movement regulation and support a model in which SNr neurons promote movements through disinhibition and suppress movements through firing-rate increases, acting on the same downstream targets.

## SNr control of brainstem postsynaptic neurons

**Extended Data Fig. 6:**
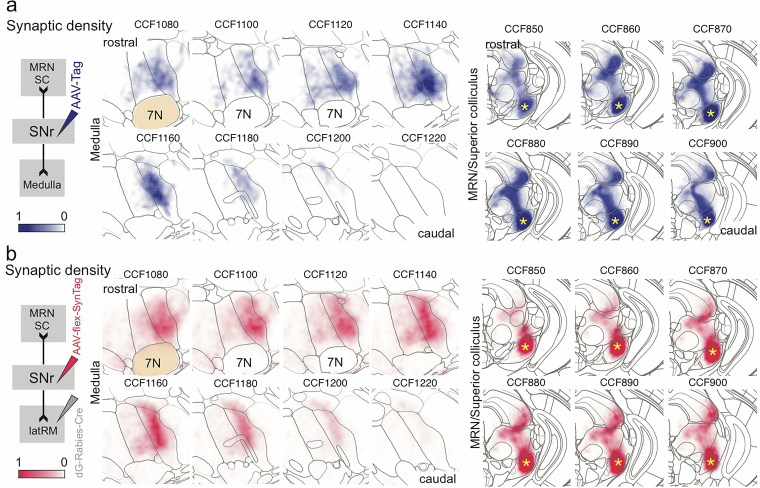
Anatomical output of SNr to latRM, MRN and SC. **a**, (left) Schematic representing viral injection scheme of a fluorescent protein (Tag) in the SNr to visualize projections to the brainstem (n = 3, Allen Connectivity map data). (centre) Mean Tag density across mice in CCF levels encompassing the lateral medulla. Sections are spaced 200 micrometres. Top is dorsal and right is lateral. 7 N: Facial nucleus. (right) Mean Tag density across mice in CCF levels encompassing the midbrain reticular nucleus (MRN) and superior colliculus (SC). Sections are spaced 100 micrometres. Top is dorsal and right is lateral. Yellow asterisk: SNr. **b**, (left) Schematic representing viral injection scheme of a Cre-dependent synaptophysin fused fluorescent protein tag (SynTag) in the SNr to visualize collaterals of SNr>latRM projection neurons expressing Cre through retrograde targeting via G-deleted Rabies (n = 3). (centre) Mean Tag density across mice in CCF levels encompassing the lateral medulla. Sections are spaced 200 micrometres. Top is dorsal and right is lateral. 7 N: Facial nucleus. (right) Mean Tag density across mice in CCF levels encompassing the MRN and SC. Sections are spaced 100 micrometres. Top is dorsal and right is lateral. Yellow asterisk: SNr. Note that caudal lateral SNr projects to latRM, MRN and SC and SNr>latRM projection neurons collateralize in the MRN and lateral SC.

**Extended Data Fig. 7:**
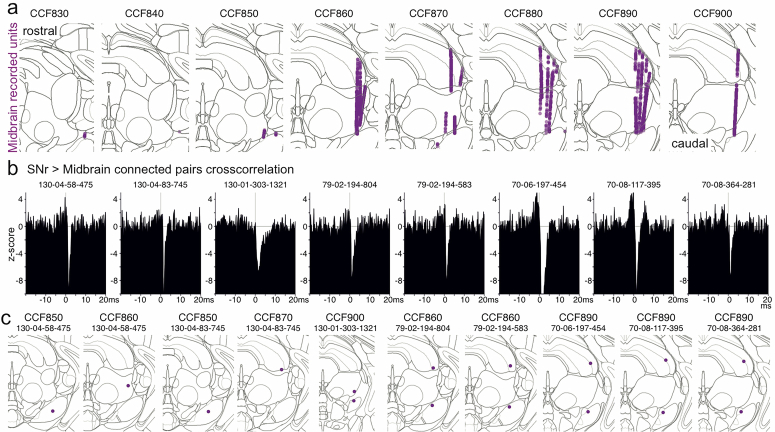
Identification of in vivo connected pairs between SNr and midbrain. **a**, Allen CCF registered location of the neurons recorded in the Midbrain along the rostro-caudal levels (displayed at 100 micrometre intervals) of the midbrain reticular nucleus (MRN) and superior colliculus (SC), here referred to as midbrain for simplicity. **b**, Z-scored cross-correlogram of the 8 identified putatively connected SNr to Midbrain neuron pairs showing cross-correlation of spike times between pre and postsynaptic neuron with minimum cross-correlation at less than 2 ms, showing strong inhibition of the postsynaptic Midbrain neuron by spikes of the presynaptic SNr neuron as a feature of all identified pairs. **c**, Respective locations of the presynaptic SNr neuron and postsynaptic Midbrain neuron for each of the 8 identified connected pairs. Note the resemblance of the locations of the Midbrain neurons to the location of the synaptic terminals from the latRM projecting SNr neurons in the midbrain shown in Extended Data Fig. 6. Source Data

Deep brain optogenetic perturbations offer the possibility to investigate the role of neuronal activity in behaviour but cannot be used to impose complex activity patterns in single neurons in vivo. We therefore next sought to determine how the complex activity of SNr neurons impacts the firing of postsynaptic neurons in the brainstem in vivo, as movement is executed. Owing to the spatial incompatibility of probe trajectories, it was not feasible to perform combined SNr and latRM recordings in freely moving mice. However, previous research suggests that caudal SNr neurons also target other nuclei in the brainstem, for example, the midbrain reticular formation and superior colliculus^29,30,42^. Exploring anterograde tracing experiments from the Allen Institute for Brain Science connectivity mapping project (https://connectivity.brain-map.org/; Methods) confirmed that, in addition to their projections to the latRM, axons from caudal lateral SNr neurons also establish branches in the midbrain (Extended Data Fig. 6a). Furthermore, our own combinatorial retrograde and anterograde tracing revealed that SNr>latRM projection neurons collateralize in the midbrain reticular nucleus and lateral deep superior colliculus (Extended Data Fig. 6b). Our Neuropixels probe trajectories targeting the SNr also traversed some regions of the midbrain area targeted by SNr projections (Extended Data Fig. 7a), giving us the rare opportunity to simultaneously record the activity of presynaptic SNr neurons and their putative recipient brainstem partners.

**Fig. 5:**
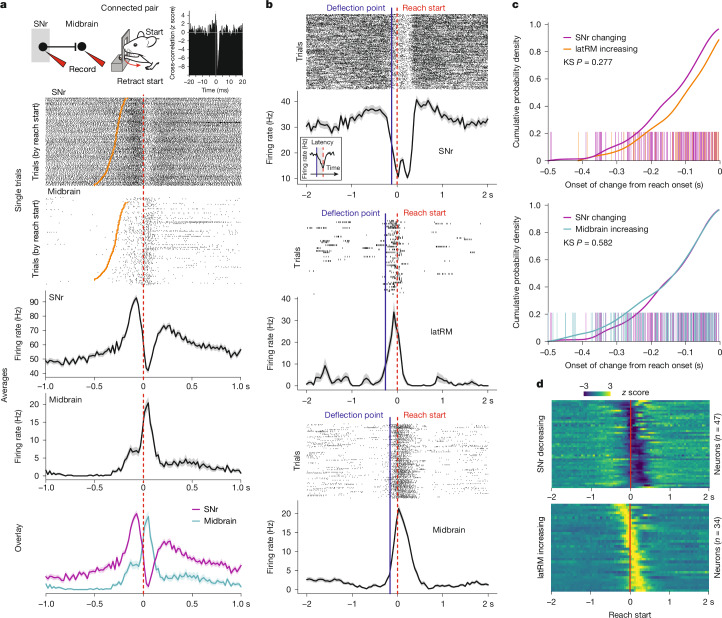
SNr control of brainstem postsynaptic neurons. **a**, Schematic of simultaneous recording from an SNr neuron and its postsynaptic midbrain neuron during the forelimb task (top left). Top right, *z*-scored cross-correlogram showing the cross-correlation of spikes between the presynaptic neuron and the postsynaptic neuron with the minimum at <2 ms, showing inhibition of the postsynaptic neuron by spikes of the presynaptic neuron. Middle, single-trial spike raster for the presynaptic (SNr) neuron and the postsynaptic midbrain neuron aligned to the retraction start (red dashed line) and sorted by reach start (orange line) time. Bottom, the mean ± s.e.m. spike rates from these trials for the presynaptic (SNr) and the postsynaptic midbrain neuron aligned to the retraction start; an overlay is shown below. Note the correspondence between the pause of the presynaptic SNr neuron and the increase in firing in the postsynaptic midbrain neuron aligned to retraction. **b**, Three example neuron (SNr, latRM and midbrain) spike raster plots, and the mean ± s.e.m. spike rates aligned to the reach start overlaid with the detected onset of a statistically significant decrease in firing for the SNr neuron and increases in firing for the latRM and midbrain neurons, as used in **c**. **c**, The cumulative probability density of onset times and overlaid single-neuron onset time rug plots with respect to reach start time for neurons displaying a statistically significant change in firing (two-sided Mann–Whitney *U*-test; Methods) before the reach onset in the SNr, an increase in firing in the latRM (top) and an increase in firing in the midbrain (bottom). This illustrates the similarity between the unfolding of neuronal activity before the movement onset in the SNr and two SNr-recipient brainstem regions. KS, Kolmogorov–Smirnov test. **d**, The *z*-scored average firing rate of SNr neurons displaying a statistically significant decrease in firing before the reach onset and latRM neurons displaying a statistically significant increase in firing before the reach onset. Source Data

**Extended Data Fig. 8:**
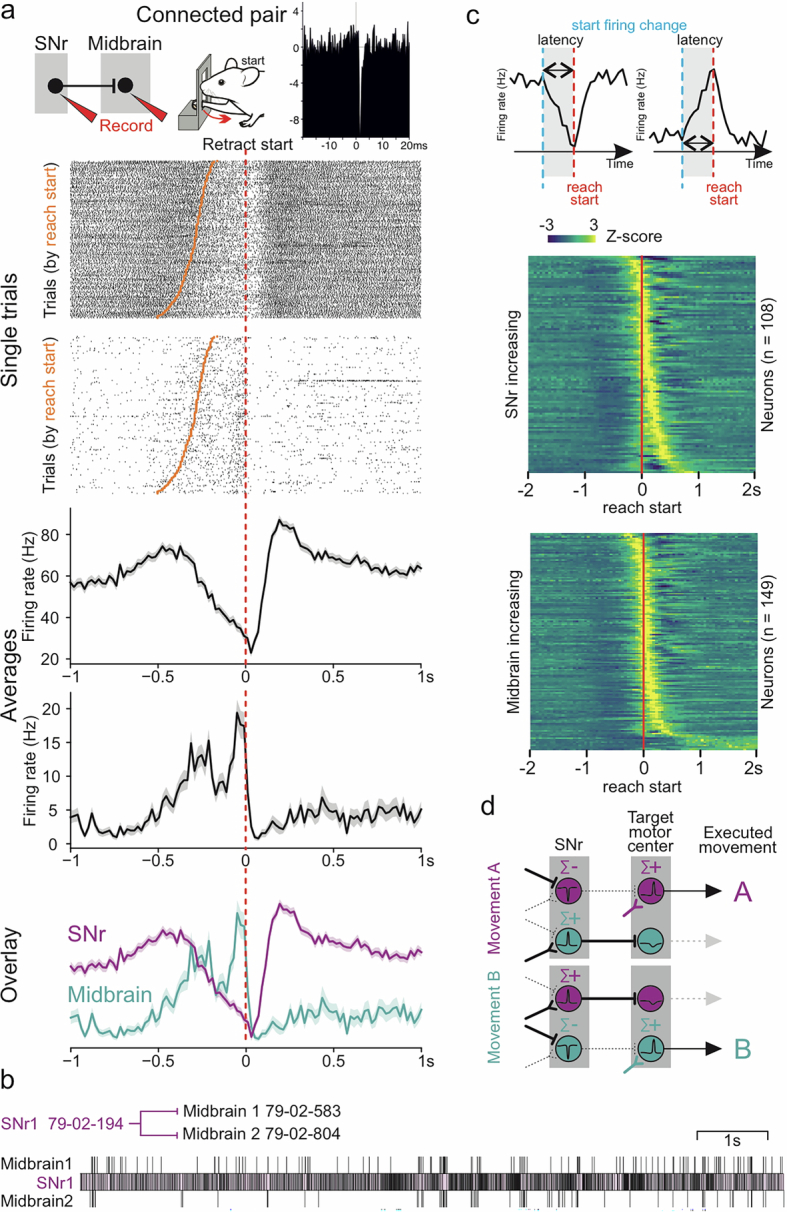
Connectivity between SNr and brainstem targets. **a**, Simultaneous recording from an example SNr neuron and its postsynaptic Midbrain neuron during the forelimb task (top left). Z-scored cross-correlogram showing cross-correlation of spike times between pre and postsynaptic neuron with minimum at less than 2 ms, showing inhibition of the postsynaptic neuron by spikes of the presynaptic neuron (top right). Single trial spike raster for the presynaptic (SNr) neuron and the postsynaptic (Midbrain) neuron aligned to retraction start and sorted by reach start time (middle). Average spike rates with standard error of the mean from these trials for the presynaptic (SNr) neuron and the postsynaptic (Midbrain) neuron aligned to retraction start with overlay shown below. **b**, Example raster plot of one SNr neuron (middle) that was identified to have two putatively postsynaptic Midbrain neurons (top and bottom) highlighting the striking correspondence between spiking in Midbrain neurons and pauses in firing of the presynaptic SNr neuron. Interestingly, not all pauses in the SNr neuron corresponded to increases in the Midbrain neurons. **c**, Schematic indicating the detection of the change in firing rate during the forelimb reaching task of SNr, latRM and Midbrain neurons (top). Briefly, first, for every neuron a stringent search was employed to identify the earliest 20 ms time-bin in the 500 ms preceding the onset of reaching which showed a statistically significant modulation from baseline firing of the neuron (two-sided Mann-Whitney test with p-value ≤ 0.001). Subsequently, rolling back from this time-bin, a lenient search was used to identify when each neuron exhibited the first 20 ms time-bin with firing rate significantly different from its baseline (two-sided Mann-Whitney test with p-value ≤ 0.05) to identify the first statistically significant deflection in firing rate of the neuron (see Methods for details on the statistical tests used to detect modulation). Heatmaps of z-scored average firing rate of SNr neurons (middle) and Midbrain neurons (bottom) with positive modulation preceding the reach onset. **d**, Model for the control of forelimb movement mediated by the basal ganglia output structure, SNr, via target motor centres in the brainstem. The schematic summarizes the activity of SNr neurons and their downstream targets during the execution of movement A (top) and movement B (bottom). Single SNr neurons show forelimb movement specific pauses and increases in firing which exert a disinhibition or suppression of their downstream motor targets respectively, thereby licensing different motor programs to be executed contingent on excitatory drive to the motor centres (see Discussion). Source Data

We used spike timing statistics to identify putative monosynaptic inhibitory connections between SNr and midbrain neurons (Methods and Extended Data Fig. 7b). Across all of the recorded mice, we identified select connected pairs as shown by their cross-correlograms, indicating inhibition of the postsynaptic midbrain neuron after spiking of the presynaptic SNr neuron (Extended Data Fig. 7b). In agreement with the anatomical distribution of axonal projections from the caudal lateral SNr, postsynaptic neurons were located in the midbrain reticular nucleus and lateral superior colliculus (Extended Data Fig. 7c), in which neuronal correlates of forelimb control have been identified in primates^43,44^. At the level of single connected pairs, we identified postsynaptic neurons displaying marked increases in firing aligned to phases of the movement (Fig. 5a and Extended Data Fig. 8a). These increases in the firing rate unfolded during the pause of their presynaptic SNr partner at a timescale relevant for behaviour (Fig. 5a and Extended Data Fig. 8a). To generalize this finding across other identified connected pairs, we visualized simultaneous neuronal spiking of pre- and postsynaptic neurons and observed again coincidence of presynaptic pauses with postsynaptic increases (Extended Data Fig. 7d). For neurons tuned to recorded movements in the task, we analysed noise correlation, a measure of single-trial firing co-variability. Noise correlation was negative at the time of peak mean activity of the midbrain neuron (correlation coefficient: −0.29 (Fig. 5a) and −0.35 (Extended Data Fig. 8a)), consistent with the idea that more pronounced pauses in the SNr correspond to more pronounced increases in downstream neurons.

While highly informative, finding connected pairs in vivo that are tuned to behaviour is extremely low throughput. Thus, to generalize these findings at the population level, we examined neuronal dynamics in the SNr and target regions before movement was executed. If SNr neurons shape neuronal activity in target regions for the production of forelimb movement, changes in neuronal activity should unfold similarly in time across neuronal populations in the SNr and the here-studied SNr-recipient brainstem areas, namely the latRM and midbrain reticular formation. We analysed neuronal activity in the SNr and midbrain using the dataset already described in this paper and an independent dataset of latRM silicon probe and Neuropixels recordings, partly composed of already published data^35^. Single neurons in the latRM and midbrain reticular formation displayed sparse increases in firing rate locked to movement onset, contrasting with the neuronal activity patterns that we observed in SNr neurons (Fig. 5b), confirming previous work relating neuronal activity in these regions to forelimb movement execution^26,35,43,44^. To address our question, we determined the first statistically significant changepoint across trials in the firing of single SNr, midbrain and latRM neurons, focusing on the 500 ms before the reach onset (Fig. 5b, Methods and Extended Data Fig. 8c). For each region, we then computed the cumulative distributions of single-neuron changepoints in time preceding the reaching onset (Fig. 5c). We found that changepoints preceded movement in temporally indistinguishable patterns between SNr, midbrain and latRM neurons (SNr versus midbrain, Kolmogorov–Smirnov *P* = 0.582; SNr versus latRM, Kolmogorov–Smirnov *P* = 0.277). Furthermore, the *z*-scored average activity of pausing and increasing SNr neurons, as well as of the midbrain and latRM neurons increasing their firing rate, demonstrated temporal similarity in modulation between regions before movement (Fig. 5d and Extended Data Fig. 8c). Together, these data provide strong support for the notion that SNr neurons exert bidirectional control over their postsynaptic partners to control movement.

## Discussion

Understanding the dynamic firing patterns of basal ganglia output neurons and their links to behaviour has been a long-standing question in neuroscience. Past progress relied on the study of movements allowing great experimental control^9,45^ but hindered generalization to behaviours composed of flexible sequential movements and a systematic analysis of firing changes in relation to different movements. Here we took advantage of skilled forelimb behaviour in mice owing to its complex nature, while being divisible into distinct phases and movements^46^. Such a complex yet natural and sequential task was essential to reveal that SNr neurons license movements through disinhibition of downstream targets and provide suppression through firing increases for movements different from the pause-related one. Each of these neuronal responses is temporally aligned with executed movement, supporting a model in which a single SNr neuron provides both movement-permissive and movement-repressive signals to its postsynaptic downstream partners (Extended Data Fig. 8c; shown for the example of movements A and B).

We found that SNr neurons exhibit multiphasic firing dynamics, with decreases and increases during the sequential execution of forelimb movements. Our modulation index analysis demonstrates that every SNr neuron presents its own precise decreases and increases in spiking in relation to different movements. Moreover, we reveal an emerging functional logic: many SNr neurons that decreased firing during reaching and/or retraction (mostly driven by proximal forelimb muscles) exhibited firing increases during food handling and manipulation movements (involving distal forelimb musculature), and the reverse, echoing the functional organization of neurons in the lateral medulla^26,35^. We hypothesize that dynamic firing changes are linked to the control of a coordinated set of muscles or the angular displacement of a specific set of joints, temporally coordinating the diversity of movements generated through connected brainstem neurons^26,35^. In agreement, we found that specific variations of the executed movements are reflected in underlying firing changes of specific SNr neurons as predicted by the observed granularity of their modulation to task time windows. These findings are incompatible with a delayed-lines model of basal ganglia function at the timescale of the here studied movements^39^. Instead, they support the view that the specific connectivity matrix from the different basal ganglia nodes and other inputs to SNr neurons^37,41,47^ are instrumental in shaping SNr movement-specific firing changes.

The granularity of observed SNr firing patterns in relation to executed movements appears similar to the one described for striatal projection neurons, despite the strong synaptic convergence from striatum to basal ganglia output^27,37^. One interesting possibility is that context-dependent recruitment of different striatal ensembles—for example, in situations entailing different goals or motivation—can engage similar SNr ensembles through convergence. Such a mechanism would ultimately enable context-dependent movement through the same precise motor outputs^29,30^, while allowing for flexible and learning-malleable pathways through the basal ganglia to contribute to movement timing and concatenation^7,33^. Furthermore, while striatal projection neurons exhibit sparse movement-related firing increases from a silent baseline^7,8,48,49^, SNr neurons exhibit bidirectional firing changes with a high dynamic range, impacting downstream neurons through both release and repression.

Our activity analysis of SNr-recipient brainstem neurons is in agreement with the notion that SNr output firing rate changes profoundly and precisely impact motor output centres. SNr neuron pauses align to firing increases in the connected brainstem neuron. However, we observed variable timing between increases of postsynaptic brainstem neurons and pauses of SNr presynaptic neurons. We hypothesize that firing increases of SNr-recipient brainstem neurons are driven by excitatory inputs from other sources, including the cortex (Extended Data Fig. 8d). Thus, convergent inputs to movement execution centres by extrapyramidal and pyramidal inputs are essential to determine movement specification. In this model, decreasing SNr firing licenses movement execution generated by excitatory synaptic inputs to movement-promoting postsynaptic brainstem neurons. As the brainstem also has inhibitory neurons, SNr neuron firing increases might also facilitate movement execution, depending on the role of inhibitory brainstem neurons in movement regulation. Support for a movement-aligned SNr code including movement-suppressive roles for SNr firing increases is also provided by our combined optogenetic perturbation and in vivo recording experiments of SNr and postsynaptic neurons. In these experiments, we observed a strong correlation between induced optogenetic effects on the SNr firing rate and movement execution. Although more refined subpopulation-specific perturbation experiments are not technically feasible due the diverse responses of single SNr neurons and their deep-brain location below dopaminergic neurons, our findings suggest that single SNr neurons dynamically disinhibit or overinhibit postsynaptic neurons to facilitate or suppress a specific movement. The work therefore helps to resolve long-standing controversies on the motor function of observed firing-rate increases in basal ganglia output^19,40,50–52^.

Our work supports a model in which the activity of SNr neurons together tiles movement space. In this space, individual SNr neurons display selectivity with a code that is temporally precise and movement specific for both decreases and increases at an extremely high level of granularity, thereby dynamically combining different signals over time (Extended Data Fig. 8d). Key to our discovery was the careful analysis of a complex yet decomposable behaviour. Our findings suggest that the basal ganglia hierarchy might have evolved to aid the emergence of this remarkable movement selectivity, a principle that might generalize to SNr neurons related to cognitive or emotional variables^28^. Our work harmonizes the understanding of basal ganglia output signalling into one coherent framework and provides an essential steppingstone to understand functional decay in movement disorders such as Parkinson’s disease.

## Methods

### Animals

Experiments were carried out in male and female mice (C57BL/6J background, wild type, *Rbp4-Cre* (MMRRC, 031125-UCD) and *vGAT-Cre* (JAX, 028862)) maintained on a mixed genetic background (129/C57BL6), aged 2–6 months at the start of the experiments. The mice were maintained at 22 ± 1 °C) at relative humidity ranging from 46–65% and under a 12 h–12 h light–dark cycle. Transgenic experimental animals were heterozygous from a backcross to C57BL6. They originated from different litters, were randomly allocated to experimental groups and were identified by earmarks. All of the procedures pertaining to housing, surgery, behavioural experiments and euthanasia were approved by the Cantonal Veterinary Office Basel-Stadt and performed in compliance with the Swiss Veterinary Law guidelines.

### Viral tools

The following adeno-associated viruses (AAVs) and rabies viruses were used for anatomical and functional experiments: AAV-flex-SynGFP (referred to as AAV-flex-SynTag)^53^, CVS-N2c-nl.mCherry-FlpO (referred to as rabies-nTag)^54^ (Addgene, 172378), SiR-N2c-iCre (referred to as rabies-Cre)^55^, AAV-EF1a-double floxed-hChR2(H134R)-EYFP (Addgene, 20298), AAV-Ef1a-DIO-ChRmine-mScarlet (Addgene, 130998), AAV-iCre-H2B-GFP, AAV-FRT-ChRmine-p2a-oScarlet, AAV-flex-FlpO-H2B-V5 (all generated as described previously)^26^. To infect neurons through local infection, a 2.9 serotype plasmid was used for production. For retrograde targeting of neurons by means of axonal infection, either rabies virus for anatomical experiments or AAV2.11^56^ for functional experiments was used. All AAVs used in this study were produced according to standard protocols. Genomic titres for AAVs were between 1 × 10^12^ and 1 × 10^14^ genome copies per ml, while for rabies between 1 × 10^7^ and 1 × 10^8^ genome copies per ml.

### Surgical procedures

Buprenorphine (Temgesic, 0.1 mg per kg) was applied subcutaneously as pre-emptive analgesia half an hour before the beginning of surgery. Mice were anaesthetized with 2–3% isoflurane using oxygen as a gas carrier. Once deeply anaesthetized, the mice were transferred to the stereotaxic frame under 1–2% isoflurane. Anaesthesia was kept constant by regulating the isoflurane concentration. Surgical equipment was disinfected, and a heating pad was used during the surgical procedures to avoid body temperature dropping. Eyes were protected from dehydration with ocular gel. Mice were injected with a mixture (50:50) of lidocaine (10 mg per kg) and ropivacaine (Naropin, 3 mg per kg) in the area of the surgery to reduce post-operative pain. Once anaesthetized, the skin was shaved and disinfected. After surgery, buprenorphine (0.1 mg per kg) was applied subcutaneously on the day of the surgery, followed by subcutaneous injection of meloxicam (5 mg per kg) at awakening to ensure analgesia and for the next 2 days at an interval of 24 h or administration of carprofen (10 mg per kg) in the drinking water from the day preceding surgery to 2 days after. Application of viruses, implantation of electrophysiological recording probes and implantation of optic fibres were directed to the target brain regions using high-precision stereotaxic instruments (Kopf Instruments, Model 1900). Stereotaxic coordinates for brain injections are defined as anteroposterior (AP), mediolateral (ML) and dorsoventral (DV) (approximate injection volumes: 20–100 nl), taking bregma or lambda as a reference for the AP and ML axes (SNr: −3.6 mm AP from bregma, 1.6 mm ML, 4–4.5 mm DV from dura mater; latRM: −1.95 mm AP from lambda, 1.5 mm ML, 4.5 mm DV from dura mater; PPN: −0.5 mm AP from lambda, 1.1 mm ML, 3 mm DV from dura mater).

### Immunohistochemistry and microscopy

After termination of in vivo experiments, mice were euthanized, and brains and spinal cords were collected for histological processing. In brief, animals were anaesthetized with a ketamine–xylazine solution and transcardially perfused with PBS, followed by a solution containing 4% paraformaldehyde in PBS. The brain and spinal cord were dissected, post-fixed overnight in 4% paraformaldehyde and incubated in 30% sucrose (w/v) in PBS for at least 2 days before cryopreservation. Coronal brain tissue sections were cut on a Cryostat at a thickness of 80 μm. Floating sections were collected in sequential order into individual wells and incubated for 1 h in blocking solution (1% BSA, 0.2% Triton X-100, PBS). Primary antibodies were then applied in blocking solution and incubated for 1–3 days at 4 °C. Fluorophore-coupled secondary antibodies (Jackson or Invitrogen) were applied to floating sections after extensive washing and incubated for 1 day at 4 °C. The sections were then washed and mounted with anti-bleach preservative medium on slides in sequential rostrocaudal order. Primary antibodies and respective dilutions used in this study were as follows: chicken anti-GFP (1:2,000, Invitrogen, A10262), rabbit anti-RFP (1:5,000, Rockland, 600-401-379), chicken anti-TH (1:500, Neuromics, CH22122), goat anti-ChAT (1:500, Millipore, AB144P). The following secondary antibodies were used all diluted 1:1,000: donkey anti-rabbit Cy3 (Jackson Immuno Research, 711-165-152), donkey anti-goat Cy5 (Invitrogen, A-21447), donkey anti-chicken 488 (Jackson Immuno Research, 703-545-155), donkey anti-chicken Cy5 (Jackson Immuno Research, 703-605-155), donkey anti-goat 488 (Invitrogen, A-11055). For low-resolution overview imaging, slides were scanned with an Axioscan light microscope (Zeiss). For higher-resolution imaging used for anterograde tracing (Extended Data Fig. 7b), we used the Axio Imager M2 microscope (Zeiss) with a Yokogawa CSU W1 dual-camera T2 spinning-disk confocal scanning unit.

### Behavioural experiments

For the pellet reaching and handling task, food-restricted mice were placed into a custom-made chamber containing a slit and trained to protrude the arm through the slit, reaching for a food reward. The body weight of mice and food consumption was monitored daily to not drop below 80% of the baseline weight. Mice were encouraged to use the forelimb for reaching trials by placing food pellets at a consistent position outside the slit and were trained for 5–10 days. All mice recorded produced stereotyped forelimb movement kinematics after training (Fig. 1b).

For the lever-pressing task, water-restricted mice were placed into a custom-made chamber containing a slit and were trained to protrude their arm through the slit, to press a custom-made lever delivering a digital signal to synchronize with the rest of the equipment. The task was programmed using the visual reactive programming language Bonsai^57^. The visual signal was delivered using a small blue LED. Water rewards were delivered through a blunt feeding needle (FST, 18061-10) and a solenoid valve (The Lee Company, LHDA0531115H). Mice were trained for 2–4 weeks in total, with a 1-day break minimally every 14 days. After training, all of the mice responded to the visual signal with a lever press in the response window as seen in the distribution of the press latency and ethograms (Fig. 4c,e and Extended Data Fig. 5b). The body weight of mice and water consumption was monitored daily to not drop below 80% of the baseline weight. Videos were recorded from below and side for pose estimation with Basler cameras (Ace 2 series with Pylon software and interfacing with Arduino IDE) for the lever-pressing and pellet-retrieval tasks.

### Electrophysiological recordings

To perform in vivo extracellular electrophysiological recordings, Neuropixels probes (IMEC, NP1.0 or 2.0)^58,59^ were implanted in the midbrain to target the SNr. Before probe implantation, Neuropixels probes were mounted onto 3D printed fixtures (ATLAS Neuroengineering)^60^. We confirmed correct probe placement and location of recording sites after termination of experiments, using immunohistochemistry and probe coating with a thin layer of DiI (Invitrogen) and all probes implanted had units putatively isolated in the SNr (Fig. 1c). For Neuropixels probes, we used the SpikeGLX software to record electrophysiological data synchronized with camera timestamps. When applicable, task-related signals (cue, lever, reward valve) and laser pulse signals were collected using the National Instruments PXIe-6341 multifunction IO module through the BNC-2110 breakout box with the National Instruments PXIe/PCIe-8281 controller module.

### Optogenetic perturbation experiments

**Extended Data Fig. 9:**
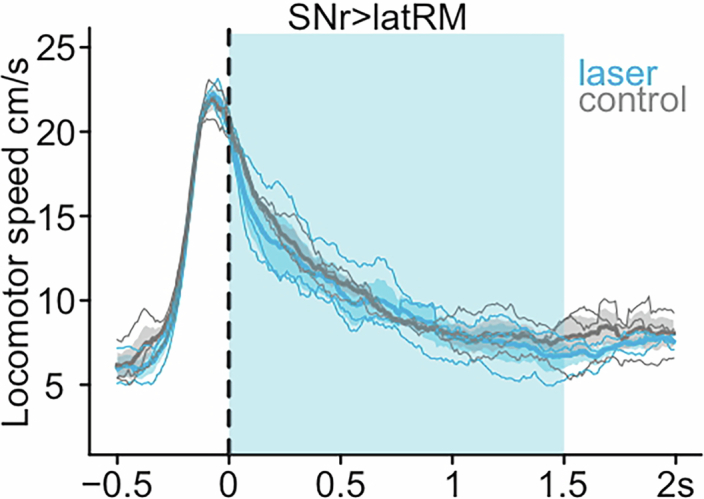
Optogenetic activation of SNr neurons projecting to latRM does not halt locomotion. Effect of optogenetic activation of SNr neurons projecting to latRM on locomotor speed (n = 3) (Fig. 4d, Extended Data Fig. 4). Optogenetic perturbation did not alter locomotor speed, despite inducing a delay to press the lever in the lever press task (Extended Data Fig. 4). Source Data

Optogenetic stimulation was performed using a PlexBright Radiant Optogenetic Stimulation System (Plexon) in combination with lasers (Cobolt 06-MLD, 473 nm; Cobolt 06-DPL, 532 nm). Light was delivered through a patch cord and rotary joint (Doric Lenses) connected to the animal’s optic fibre (Doric Lenses C60 or FLT). The laser intensity was measured at the beginning of every session using an optical power meter (Thorlabs) on the tip of an optic fibre with the same characteristics as the one implanted to ensure consistent stimulation power. Optogenetic stimulation was provided at the cue offset (100 ms from presentation) on 40–50% of randomly selected trials. A continuous light pulse was used when stimulating the PPN>SNr neurons as described previously^41^. In the case of the optogenetic activation of latRM projecting SNr neurons, a 100 Hz 50% duty cycle signal was used to drive the laser to activate the SNr neurons. Fibre tip powers used in these experiments ranged between 1 mW and 20 mW. For the stimulation of the PPN>SNr neurons, we injected AAV2.9-Ef1a-DIO-ChRmine-mScarlet or AAV2.9-EF1a-double-floxed-hChR2(H134R)-eYFP in the PPN of *Rbp4-cre* mice. In the case of the stimulation of the SNr>latRM neurons, we injected AAV2.11-iCre-H2B-GFP into the latRM and AAV2.9-Ef1a-DIO-ChRmine-mScarlet in the SNr of WT mice or AAV2.11-flex-FlpO-H2B-V5 in the latRM and AAV2.9-FRT-ChRmine-p2a-oScarlet in the SNr of *Vgat-cre* mice. This dual strategy was motivated by previous findings reporting paradoxical silencing of SNr neurons after optogenetic activation due to local inhibitory collaterals^61^. To control for the effects of SNr>latRM neuron stimulation, we performed the same perturbation as in the lever-pressing task in an open-field arena in a closed-loop with locomotion using Bonsai^57^. Specifically, light was delivered when the mouse crossed a locomotor speed (centroid speed) threshold of 10 cm s^−1^ for 150 ms. The results of these experiments are shown in Extended Data Fig. 9, revealing no significant effect on locomotor speed (for comparison see fig. 6f in ref. ^41^).

### Quantification and data analysis

#### Anatomical reconstructions and data analysis

Maximum-intensity projections of coronal brain sections obtained from a confocal microscope using a ×20 objective (anterograde tracing) or from a light microscope with a ×5 objective (nucleus segmentation) were manually linearly registered and aligned to the Allen CCF using an adapted version of AP_histology (https://github.com/petersaj/AP_histology).

Nuclei in fluorescence images were detected using a custom implementation of stardist in ImageJ (https://imagej.net/plugins/stardist) and their centroid coordinates were transformed into CCF coordinates for visualization over contour plots of the CCF annotation volume. For each CCF level, nuclei from the preceding and following 50 μm AP were gathered and plotted and their 2D density estimated at each pixel on a CCF level using kernel density estimation (Extended Data Fig. 1b). Projection density was quantified starting from a thresholding step where the SynTag signal was binarized from the background and manually curated to remove autofluorescent artifacts from immunohistochemical processing. The density of SynTag-positive pixels falling in each CCF voxel (resolution of 80 × 10 × 10 μm, AP × DV × ML) was then computed thereby generating a 3D volume in CCF space of SynTag density. The obtained density was filtered using a Gaussian kernel with a s.d. of 2,3,3 (AP × DV × ML) and truncated at 2 s.d. on each dimension. The obtained density array was then zoomed using second-order spline interpolation to reach a final voxel size of 10 × 10 × 10 μm (AP × DV × ML) and normalized to between 0 and 1. Projection density was averaged across mice, scaled and then displayed over equally spaced CCF coronal levels, plotting the respective CCF annotation volume as a contour plot (Extended Data Fig. 7).

#### Analysis of Allen Brain Institute BrainMap data

To quantify brainstem projections of SNr neurons, we used experiments numbers 100141993, 175263063 and 299895444 from the Allen Brain Connectivity atlas (https://connectivity.brain-map.org/)^62^. Volumetric projection density data were downloaded as nrrd files using the Allen SDK (https://allensdk.readthedocs.io). Projection density was averaged across experiments, scaled and then visualized in equally spaced CCF coronal levels plotting the respective annotation volume as a contour plot.

#### Behavioural analysis in the forelimb reaching task

For analysis of forelimb movements executed during the forelimb task, we applied deep-neural-network-based markerless pose estimation using DeepLabCut^63^ coupled with high-speed videography of the bottom view of the mouse at 100 fps to track the moving hand and the slit. We used a DeepLabCut model trained on frames of different videos of mice from the bottom view over many similar behavioural experiments^35^. Obtained predictions were median-filtered with a filter size of 5 or filtered using sosfiltfilt in Python with an order of 4 and frequency of 15. Reaches were detected as peaks in the position of the hand over the slit with a prominence threshold (slit crossings) based on the two-dimensional position of the hand and slit as seen from the bottom view. The retraction start was defined as the moment of maximum extension (also corresponding to reach stop), and the reach start was detected by rolling back from the retraction start in a time window of 0.5 s to the moment when the hand velocity along the main reach direction decreased below a threshold of 1 pixel per second. The retraction stop was detected rolling forward from the retraction start as the moment when the hand velocity along the main reach direction increased above a threshold of −1 pixel per second. We identified isolated reaches as those separated from previous and next reach by a minimum of 0.75 s. Average velocity profiles were computed for each mouse and then averaged across mice for Fig. 1b. Handle start, manipulation start and stop were manually detected as the hand to mouth movement that mice perform to start consuming the pellet, the downwards movement away from the mouth during handling that precedes pellet manipulation and regripping, and an upward movement towards the mouth that precedes chewing, respectively^38^. To compute modulation during task time windows, we isolated select time windows, overall capturing the sequence of produced movements in the task (Supplementary Video 1): (1) reach start, from −150ms to +25 ms from the detected reach start^64^; (2) reach, from reach start to retraction start; (3) reach distal, from slit crossing to retraction start, to capture the lateral forelimb movement towards the pellet; (4) retraction start, a 50 ms window starting at the retraction start encompassing finger closure; (5) retraction, from retraction start to retraction stop; (6) handle start, the 100 ms window centred at handle start; (7) manipulation start, the 50 ms after the annotated manipulation start timepoint; (8) manipulation, from start to stop; and (9) manipulation stop, the 50 ms after the annotated manipulation stop timepoint. The repeated reach trials were identified as those pairs of detected reaches that occurred within 0.3–0.6 s of each other, comparing the detected retraction start timestamps (Fig. 2d and Extended Data Fig. 2d). To stratify trials in which different movement phases were selectively altered we used either kinematics tracking or manual annotation. To identify trials with different reach duration, we identified in each animal the trials that lay between the 1st and 25th percentile of reach durations (short reaches) and 73rd to 97th percentile (long reaches) (Supplementary Video 2). We did not use the extremes of the data to avoid capturing idiosyncratic trials. Abbreviated reaches were defined as those trials in which the arm extended maximally 0.45 cm past the slit on the major movement axis and 0.25 cm on the minor axis and thus did not reach the pellet (Supplementary Video 2). Reach trials followed by pellet retrieval were identified manually. These trials, in which the reach was followed by handling, were contrasted to the remaining detected isolated reaches in an analysis comparing the activity between these trial types.

#### Electrophysiological data analysis

All data were processed using the ecephys spike sorting modules for SpikeGLX (https://github.com/jenniferColonell/ecephys_spike_sorting). In brief, data collected from SpikeGLX were first processed using the CatGT module to apply demultiplexing corrections, removing electrical artefacts (using ‘gfix’) and high-pass filtering the data. Moreover, the edges of the synchronization pulses from the IMEC base station on which the Neuropixels data were recorded, the camera exposure pulses and laser pulses were extracted. Subsequently, using the Kilosort helper module, channels with a firing rate below 0.05 Hz were excluded as noisy channels and the channel map for the spatial location of the remaining channels was constructed using the metadata from the recordings. We used Bank 0 on the Neuropixels 1.0 to record from the ventral-most 384 channels on the probe. Subsequently, Kilosort3 was run on the data. After the sorting, TPrime module helped to synchronize all of the datastreams precisely, with the IMEC base station recording as the reference time stream, using the synchronization pulse recorded on both the multifunctional IO device and the IMEC base station. To align a probe tract to anatomy, we registered the probe tract identified by DiI signal to mark the Neuropixels probes, to the Allen CCF (https://github.com/petersaj/AP_histology). Using the ephys alignment tool from the International Brain Laboratory (https://github.com/int-brain-lab/iblapps/wiki), we aligned the electrophysiological features from the data to the anatomical landmarks to obtain the precise probe trajectory and channel locations in CCF coordinates. We then performed manual curation of the output of Kilosort3 in Phy 2 to obtain isolated single units. Single units were identified in anatomical CCF space based on their peak channel and plotted over the closest 100 μm spaced CCF level. For SNr recordings, based on extensive previous literature concerning the tonic activity of neurons^9–20,29,31,36,45,65–69^, we isolated neurons with a mean spike rate over the entire recording session higher than 5 Hz. This resulted in a dataset of 646 units across 17 mice for the pellet-reaching task and 184 neurons across 5 mice for the lever-pressing task with optogenetic perturbation. For the remaining midbrain and latRM electrophysiology data, all curated single units were included for analysis (2,197 neurons across 17 mice in the remaining midbrain and 709 neurons across 8 mice in the latRM). For the latRM, neuronal recordings from four mice were part of a previously published dataset^35^, while four mice were recorded for this work. The firing rate was binned into bins of 50 ms for subsequent plotting of single-neuron PETHs. For heat maps and average firing rates across populations of SNr neurons in Figs. 2 and 3 and the related Extended Data Figs. 2 and 3, we computed the firing rate of each neuron in 5 ms bins with a Gaussian filter with a sigma of 10 and a size of 100 ms. Average firing rates in 2 s windows surrounding retraction start were concatenated across all trial types considered in the manuscript (see the ‘Behavioural analysis in the forelimb reaching task’ section) and *z*-scored for display and further analysis of the effects movement variation on neuronal activity.

#### Task event modulation

To identify SNr neurons modulating firing rate aligned to different phases of the analysed forelimb tasks, we calculated modulation during the task time windows defined in the ‘Behavioural analysis in the forelimb reaching task’ section above. For each neuron and each time window, we computed the mean spike rate during the window across *N* trials and defined a null distribution of mean firing rates computed over 1,000 groups of *N* random windows. We defined modulation as the difference between the mean spike rate during the window and the median of the null distribution of mean firing rates, normalized to the sum of these two variables, to mitigate the effects of differences in baseline spiking. To compute the statistical significance of the computed modulation, we ranked the mean spike rate in the task time window with respect to the null distribution and considered as significant only modulations higher than an absolute 0.025 threshold, with a rank either between the 0th and the 1st percentile or between the 99th and 100th percentile of the null distribution for negative and positive modulation, respectively.

#### Correlational structure of neuronal activity during repeated reach events

To evaluate the neuron-to-neuron correlational structure of neuronal activity, we correlated the average firing rates of all recorded neurons for each single mouse in a −2s to +2 s window around the relevant timestamp (for example, first retraction start or random timestamps in the session). The resulting pairwise Pearson correlation across neurons was plotted in the form of a heat map in Extended Data Fig. 2e. We then regressed the obtained pairwise neuron correlations across timestamps and obtained the slope and *R* value of the fit across pairs of timestamps to generate summary bar plots in Extended Data Fig. 2e.

#### Analysis of changes in spiking activity in movement variation trials

To discern the effects of trial type on the firing rate across all examined pairs of trials (Fig. 3a; see the ‘Behavioural analysis in the forelimb reaching task’ section), we compared firing rates of different neuronal populations to a null distribution of average firing rates computed over 1,000 random groups of trials sampled from each trial type. Given *N* trials of type 1 and *M* trials of type 2, with *N* < *M*, we generated 1,000 random groups of *N* trials, equally sampling from trial type 1 and 2. For each neuron, we computed the average firing rate for each of these 1,000 random groups, thereby obtaining a null distribution of firing rates aligned to the retraction start, and *z*-scored using the mean and s.d. obtained across all trial types considered in the Article (see the ‘Behavioural analysis in the forelimb reaching task’ section). For each neuronal population, we identified *z*-scored average firing rate windows longer than 50 ms in which the difference in firing rate in the two trial types was larger than the 99.9% confidence interval of the null distribution of average firing rates for that neuronal population. In practice, we identified data timepoints at which the average firing rate of the population in one trial type was below the 0.05th percentile and the other trial type above the 99.95th percentile of the null distribution of average firing rates for that neuronal population.

This analysis was carried out on specific populations of single units: (1) SNr neurons pausing throughout arm extension, that is, reach and reach distal time windows and not negatively modulated during handling-related time windows (handle start, manipulation start, manipulation stop, manipulation); (2) SNr neurons negatively modulated at the retraction start but not during the reach time window; (3) SNr neurons negatively modulated at the handle start but not during any proximal task time window (reach start to retract). For Extended Data Fig. 3i, we isolated a subset of neuronal population 2, the SNr neurons negatively modulated at retraction start and during at least one handling-related time window.

#### Analysis of neuronal recordings during optogenetic perturbation

To quantify the response of SNr neurons to optogenetic excitation, in each mouse, we computed the average laser-evoked and cue-evoked firing rate of each SNr neuron. Obtained arrays were concatenated together and *z*-scored for each single neuron using the mean and s.d. of firing rates in control trials. We then averaged across the recorded SNr population for each recorded mouse (Fig. 4c) and, subsequently, across mice (Fig. 4d (top)). We then performed the same operation on the lever-press rate computed in 200 ms bins. In brief, we computed the average laser-evoked and control press rates and plotted their difference as the average across mice. We also computed the fraction of trials with a lever press in the response time in control and stimulation trials for each mouse and plotted the ratio of laser over control response rates as control normalized response rate (Extended Data Fig. 5a). To further quantify behavioural effects of laser exposure we compared the distribution of press latencies in laser versus control trials using Kolmogorov–Smirnov tests on single mice and plotted the single mouse and average cumulative distribution across mice (Fig. 4e) and experiments (Extended Data Fig. 5b). To regress laser response against median increase in latency to press the lever, for each mouse we computed the time during which laser-evoked SNr population response was at least 1 s.d. above the control (s.d. of control responses).

To quantify lever-press-related pauses in laser and control trials (Fig. 4f), we isolated neurons that were negatively modulated to the press in a 200 ms window centred around the lever press in control trials. We calculated the modulation for each trial of the behaviour for each neuron as the average firing rate in the behaviour window in that trial above the baseline. For the modulation to the lever press, we used the 200 ms window preceding the presentation of the cue as the baseline. The distribution of modulation indices (MIs) obtained was compared to the distribution of MIs obtained for the same neuron from a minimum of 200 random timepoints sampled over the entire recording session or as many as those of the behaviour in a time window equal to the one used for the behaviour in question using a Mann–Whitney *U*-test. A neuron was classified as modulated when it had *P* < 0.05 and the average of the MI over all of the trials of the behaviour was smaller than 0 for negative modulation.

#### Connectivity analysis across SNr and midbrain neurons

To identify connected pairs of neurons between SNr and midbrain, we used Python and the package Neuropyxels (https://zenodo.org/records/5509776). We used the gen_sfc function with the default parameters to identify inhibitory putative monosynaptic connections using the Poisson Stark test for significance^70^ and a *P*-value threshold of 0.02. This procedure is based on convolving the cross-correlogram (computed with 0.5 ms bins) with a partially hollowed window. We searched for functional negative correlation in the cross-correlogram within 1 to 2.5 ms, from putative presynaptic neuron spike times. We then filtered obtained connections to identify the ones with a negative correlation peak within 2.5 ms in the *z*-scored correlogram, and a minimum *z*-scored correlation amplitude less than −10. *z*-Scored correlations (presynaptic to postsynaptic) were then visualized for each single connected pair in Fig. 5a and Extended Data Figs. 8b and 9a. The location of all connected pairs of neurons was also visualized in CCF space as described in the ‘Anatomical reconstructions and data analysis’ section above. To compute noise correlations between pre- and postsynaptic neuron firing during behaviour, we correlated spike rate in 20 ms bins across a window of 400 ms centred around the relevant behavioural timestamp.

#### Onset latency of neuronal activity before reach start

For each dataset (SNr, Midbrain, latRM), we analysed the activity of single neurons across all isolated reach start timestamps. Taking a timeframe of 0.5 s before reach start across trials, we identified neurons that were significantly (*P* < 0.001) modulated during any 20 ms window sliding 1 ms at a time, as compared to the baseline firing rate from −0.70 to −0.50 s before reach start (see modulation computation above). Having identified these neurons and the earliest modulated window before reaching start, we rolled back to find the earliest time window before a modulation *P* value of less than 0.05 was displayed. The start time of that 20 ms window for each neuron was considered to be the onset of a consistent neuronal activity change preceding reach onset across trials (Fig. 5b and Extended Data Fig. 8c). The distribution of onsets across neurons belonging to different brain regions or modulated positively and negatively for SNr was determined using a Kolmogorov–Smirnov test (Fig. 5c). We then plotted the *z*-scored average firing rate of single neurons detected as described above aligned to reach start (Fig. 5d and Extended Data Fig. 8c).

#### Software and statistics

All analyses were performed using custom code in Python or MATLAB as specified above. Sample sizes were not predetermined, and nonparametric statistical tests (Wilcoxon signed-rank and Kolmogorov–Smirnov tests) were always used to avoid assuming normal distributions. Probability densities were estimated using kernel density estimation with a Gaussian kernel. All *P* values are indicated either in the text or in the figure legends. Figures were prepared in CorelDRAW v.24.4. Mouse drawings were provided by E. Tyler and L. Kravitz through the SciDraw repository (www.scidraw.io) and adapted in CorelDraw.

### Reporting summary

Further information on research design is available in the Nature Portfolio Reporting Summary linked to this article.

## Online content

Any methods, additional references, Nature Portfolio reporting summaries, source data, extended data, supplementary information, acknowledgements, peer review information; details of author contributions and competing interests; and statements of data and code availability are available at 10.1038/s41586-025-09066-z.

## Supplementary information

Reporting Summary

Supplementary Video 1 Annotation of movement timepoints in the pellet reaching task. Multiple forelimb pellet reaching trials with overlaid task events as in Fig. 1b. The mouse extends the arm from inside the box to outside the slit to reach for the pellet. Grasping is temporally aligned to the onset of retraction and, after retraction stop, the mouse starts handling the food pellet. The colour code used in the video is aligned to the one shown in Fig. 1b.

Supplementary Video 2 Movement variations observed in the pellet reaching task. Multiple forelimb pellet reaching trials stratified as in Fig. 3a in short versus long duration reaches, complete reach and abbreviated reaches and reaches followed by handling or not. Note the difference in reach duration and reach start distance from slit in short versus long reaches and the absence of distal forelimb movements at retraction start in abbreviated reaches.

Supplementary Video 3 Optogenetic stimulation of SNr neurons delays lever pressing. The video shows 5 control and 5 optogenetic activation trials from one mouse seen from the bottom view. The visual cue is visible at the bottom to signal the beginning of the response time. Note the delay or absence of lever-press execution after laser exposure.

## Source data

Source Data Fig. 1

Source Data Fig. 2

Source Data Fig. 3

Source Data Fig. 4

Source Data Fig. 5

Source Data Extended Data Fig. 1

Source Data Extended Data Fig. 2

Source Data Extended Data Fig. 3

Source Data Extended Data Fig. 4

Source Data Extended Data Fig. 7

Source Data Extended Data Fig. 8

Source Data Extended Data Fig. 9

## Data Availability

Materials as well as methods used and generated are available in the key resource table in Zenodo^71^ (10.5281/zenodo.15131548). The most up to date key resource table alongside their persistent identifiers for data, protocols, and key laboratory materials used and generated in this study can be found at Zenodo^71^. Moreover, anatomical tracing data from the Allen Brain Connectivity atlas (https://connectivity.brain-map.org/) were used in this study. Any additional information is available from the corresponding author on request. Source data are provided with this paper.
