# Supplementary material for: Dynamic basal ganglia output signals license and suppress forelimb movements
Source: Nature. 2025 May 28;644(8077):749–58. doi: 10.1038/s41586-025-09066-z (PMC12367548; doi:10.1038/s41586-025-09066-z)
Supplement: Supplementary file 1 — Reporting Summary [file 41586_2025_9066_MOESM1_ESM.pdf]

nature portfolio

Corresponding author(s): Silvia Arber

Last updated by author(s): Apr 9, 2025

Reporting Summary

Nature Portfolio wishes to improve the reproducibility of the work that we publish. This form provides structure for consistency and transparency in reporting. For further information on Nature Portfolio policies, see our Editorial Policies and the Editorial Policy Checklist.

Statistics

For all statistical analyses, confirm that the following items are present in the figure legend, table legend, main text, or Methods section.

n/a	Confirmed	
☐	☒	The exact sample size (*n*) for each experimental group/condition, given as a discrete number and unit of measurement
☐	☒	A statement on whether measurements were taken from distinct samples or whether the same sample was measured repeatedly
☐	☒	The statistical test(s) used AND whether they are one- or two-sided<br>*Only common tests should be described solely by name; describe more complex techniques in the Methods section.*
☒	☐	A description of all covariates tested
☐	☒	A description of any assumptions or corrections, such as tests of normality and adjustment for multiple comparisons
☐	☒	A full description of the statistical parameters including central tendency (e.g. means) or other basic estimates (e.g. regression coefficient) AND variation (e.g. standard deviation) or associated estimates of uncertainty (e.g. confidence intervals)
☐	☒	For null hypothesis testing, the test statistic (e.g. *F*, *t*, *r*) with confidence intervals, effect sizes, degrees of freedom and *P* value noted<br>*Give P values as exact values whenever suitable.*
☒	☐	For Bayesian analysis, information on the choice of priors and Markov chain Monte Carlo settings
☒	☐	For hierarchical and complex designs, identification of the appropriate level for tests and full reporting of outcomes
☐	☒	Estimates of effect sizes (e.g. Cohen's *d*, Pearson's *r*), indicating how they were calculated

Our web collection on statistics for biologists contains articles on many of the points above.

Software and code

Policy information about availability of computer code

Data collection	Camera recording: Bonsai (v2.7), Arduino IDE (v1.8.19), Pylon(7.1.0) ; Electrophysiology: SpikeGLX (Release v20201103-phase30, Release v20230120-phase30, Release v20230411-phase30), Optogenetics: Radiant (v2.3.0, Plexon Inc.). See also Methods section for details
Data analysis	MATLAB (2022b; The Mathworks Inc.), Python3, Kilosort3.0, DeepLabCut (2.3), Phy2, Fiji / ImageJ (v2.1.0), ECephys_spike_sorting (https://github.com/jenniferColonell/ecephys_spike_sorting forked from the Allen institute spike sorting pipeline v0.2), CatGT (v2.5), Tprime (v1.8), AP_histology (https://github.com/petersaj/AP_histology), StarDist (0.3.0), Allen SDK (v2.13.1), Neuropyxels (v2.0.4, https://github.com/m-beau/NeuroPyxels), CorelDraw (v24.4). No custom algorithms were generated in this manuscript. Relevant data and example code related to the loading of specific data formats provided is available at DOI: 10.5281/zenodo.15131548

For manuscripts utilizing custom algorithms or software that are central to the research but not yet described in published literature, software must be made available to editors and reviewers. We strongly encourage code deposition in a community repository (e.g. GitHub). See the Nature Portfolio guidelines for submitting code & software for further information.

Data

Policy information about availability of data

All manuscripts must include a data availability statement. This statement should provide the following information, where applicable:

- Accession codes, unique identifiers, or web links for publicly available datasets
- A description of any restrictions on data availability
- For clinical datasets or third party data, please ensure that the statement adheres to our policy

Materials, methods used and generated are available in the key resource table in Zenodo (DOI: 10.5281/zenodo.15131548). Any additional information is available from the lead contact upon request. For the most up to date Key Resource Table alongside their persistent identifiers for data, protocols, and key lab materials used and generated in this study can be found on Zenodo.
Additionally, anatomical tracing data from the Allen Brain Connectivity atlas (https://connectivity.brain-map.org/) was used in this study.

Research involving human participants, their data, or biological material

Policy information about studies with human participants or human data. See also policy information about sex, gender (identity/presentation), and sexual orientation and race, ethnicity and racism.

Reporting on sex and gender	NA
Reporting on race, ethnicity, or other socially relevant groupings	NA
Population characteristics	NA
Recruitment	NA
Ethics oversight	NA

Note that full information on the approval of the study protocol must also be provided in the manuscript.

Field-specific reporting

Please select the one below that is the best fit for your research. If you are not sure, read the appropriate sections before making your selection.

☒ Life sciences ☐ Behavioural & social sciences ☐ Ecological, evolutionary & environmental sciences

For a reference copy of the document with all sections, see nature.com/documents/nr-reporting-summary-flat.pdf

Life sciences study design

All studies must disclose on these points even when the disclosure is negative.

Sample size	Sample sizes were accessed considering the technical requirements of each experiments. The numbers of mice and recorded cells were large and similar to those reported by previous similar studies (eg. PMID: 36608651) . Exact sample sizes are described in the figure legends and methods. Sample sizes were not pre-defined using any statistical methods. Statistical analyses were tailored to capture the variability in techniques and underlying biology.
Data exclusions	No data were excluded with satisfactory anatomical targeting of structures in all surgical procedures as described in the methods.
Replication	All experiments involved replication of the results in multiple mice as detailed in the figure legends and methods. All performed experiments included mice tested in multiple batches to avoid batch effects.
Randomization	All mice were allocated randomly to experimental groups and optogenetics experiments involved within mouse control and experimental trials.
Blinding	Investigators were not blinded. There were no experiments involving different mice for control and experimental groups. All mice performed different trials belonging to the experimental or control group and these trials were randomly selected by a computer program and the task carried out automatically. The same analysis applied to all trials which were subsequently divided into experimental and control trials for plotting.

Reporting for specific materials, systems and methods

We require information from authors about some types of materials, experimental systems and methods used in many studies. Here, indicate whether each material, system or method listed is relevant to your study. If you are not sure if a list item applies to your research, read the appropriate section before selecting a response.

Materials & experimental systems

n/a	Involved in the study
☐	☒ Antibodies
☒	☐ Eukaryotic cell lines
☒	☐ Palaeontology and archaeology
☐	☒ Animals and other organisms
☒	☐ Clinical data
☒	☐ Dual use research of concern
☒	☐ Plants

Methods

n/a	Involved in the study
☒	☐ ChIP-seq
☒	☐ Flow cytometry
☒	☐ MRI-based neuroimaging

Antibodies

Antibodies used

Primary antibodies:
Chicken anti-GFP (Invitrogen Cat# A10262), Rabbit anti-RFP (Rockland Cat# 600-401-379), chicken anti-TH (Neuromics Cat# CH22122), goat anti-ChAT (Millipore Cat# AB144P).
Secondary antibodies:
Donkey anti-rabbit Cy3 (Jackson Immuno Research Cat#711-165-152), Donkey anti-goat Cy5 (Invitrogen Cat# A-21447), Donkey anti-chicken 488 (Jackson Immuno Research Cat#703-545-155), Donkey anti-chicken Cy5 (Jackson Immuno Research Cat#703-605-155), Donkey anti-goat 488 (Invitrogen Cat# A-11055)

Validation

All primary antibodies are commercially available and have been used and validated in previous publications.
Manufacturers report all the antibodies to be used for immuno-histochemistry in mouse brain tissue. Specific validation statements where available are noted below.

Chicken anti-GFP (Invitrogen Cat# A10262):
Manufacturer demonstrated antibody specificity by detection of different targets fused to GFP tag in transiently transfected lysates tested. Relative detection of GFP tag was observed across different proteins fused with GFP in H3-GFP and p65-GFP. GFP-variant, YFP is also being detected in His-p65-YFP lysate using Anti-GFP Polyclonal Antibody (Product # A10262) in Western Blot.
Rabbit anti-RFP (Rockland Cat# 600-401-379):
Manufacturer reports to expect reactivity against RFP and its variants: mCherry, tdTomato, mBanana, mOrange, mPlum, mOrange and mStrawberry. They report that assay by immunoelectrophoresis resulted in a single precipitin arc against anti-Rabbit Serum and purified and partially purified Red Fluorescent Protein (Discosoma). No reaction was observed against Human, Mouse or Rat serum proteins.

Animals and other research organisms

Policy information about studies involving animals; ARRIVE guidelines recommended for reporting animal research, and Sex and Gender in Research

Laboratory animals

Experiments were carried out in male and female mice (C57BL/6J background, Wild type, Rbp4-Cre (RRID: MMRRC_031125-UCD) and vGAT-Cre (RRID: IMSR_JAX:028862)) 2 - 6 months of age at the start of the experiments. The mice were maintained at 22 degrees Celsius (+/- 1 degree Celsius) at relative humidity ranging from 46-65% and 12 hour light/dark cycle.

Wild animals

No animals from the wild were used.

Reporting on sex

All experiments of the study were performed in both male and female mice without distinction.

Field-collected samples

No field collected samples were used in this study.

Ethics oversight

The procedures pertaining to housing mice, surgery, behavioral experiments, recordings and euthanasia were approved by the Cantonal Veterinary Office Basel-Stadt and performed in compliance with the Swiss Veterinary Law guidelines.

Note that full information on the approval of the study protocol must also be provided in the manuscript.

Plants

Seed stocks	NA
Novel plant genotypes	NA
Authentication	NA
